# The transcriptome of the invasive eel swimbladder nematode parasite *Anguillicola crassus*

**DOI:** 10.1186/1471-2164-14-87

**Published:** 2013-02-08

**Authors:** Emanuel Heitlinger, Stephen Bridgett, Anna Montazam, Horst Taraschewski, Mark Blaxter

**Affiliations:** 1 Department of Ecology and Parasitology, Zoological Institute, Karlsruhe Institute of Technology, Kornblumenstrasse 13, Karlsruhe, Germany; 2 Institute of Evolutionary Biology, The Ashworth Laboratories, The University of Edinburgh, The King’s Buildings, Edinburgh EH9 3JT, UK; 3 The GenePool Genomics Facility, The Ashworth Laboratories, The University of Edinburgh, The King’s Buildings, Edinburgh EH9 3JT, UK; 4 Department for Molecular Parasitology, Institute for Biology, Humboldt University Berlin, Philippstrasse 13, Haus 14, Berlin, Germany

## Abstract

**Background:**

*Anguillicola crassus* is an economically and ecologically important parasitic nematode of eels. The native range of *A. crassus* is in East Asia, where it infects *Anguilla japonica*, the Japanese eel. *A. crassus* was introduced into European eels, *Anguilla anguilla*, 30 years ago. The parasite is more pathogenic in its new host than in its native one, and is thought to threaten the endangered *An. anguilla* across its range. The molecular bases for the increased pathogenicity of the nematodes in their new hosts is not known.

**Results:**

A reference transcriptome was assembled for *A. crassus* from Roche 454 pyrosequencing data. Raw reads (756,363 total) from nematodes from *An. japonica* and *An. anguilla* hosts were filtered for likely host contaminants and ribosomal RNAs. The remaining 353,055 reads were assembled into 11,372 contigs of a high confidence assembly (spanning 6.6 Mb) and an additional 21,153 singletons and contigs of a lower confidence assembly (spanning an additional 6.2 Mb). Roughly 55% of the high confidence assembly contigs were annotated with domain- or protein sequence similarity derived functional information. Sequences conserved only in nematodes, or unique to *A. crassus* were more likely to have secretory signal peptides. Thousands of high quality single nucleotide polymorphisms were identified, and coding polymorphism was correlated with differential expression between individual nematodes. Transcripts identified as being under positive selection were enriched in peptidases. Enzymes involved in energy metabolism were enriched in the set of genes differentially expressed between European and Asian *A. crassus*.

**Conclusions:**

The reference transcriptome of *A. crassus* is of high quality, and will serve as a basis for future work on the invasion biology of this important parasite. The polymorphisms identified will provide a key tool set for analysis of population structure and identification of genes likely to be involved in increased pathogenicity in European eel hosts. The identification of peptidases under positive selection is a first step in this programme.

## Background

The nematode *Anguillicola crassus* Kuwahara, Niimi et Itagaki, 1974 is a native parasite of the Japanese eel *Anguilla japonica*[1]. Adults localise to the swim bladder where they feed on blood [2]. Larvae are transmitted via crustacean intermediate hosts [3]. Originally endemic to East Asian populations of *An. japonica*, *A. crassus* has attracted interest due to recent anthropogenic expansion of its geographic and host ranges to Europe and the European eel, *Anguilla anguilla*. *A. crassus* was recorded for the first time in Europe in North-West Germany in 1982 [4], where it was most likely introduced through the live-eel trade [5,6]. *A. crassus* has subsequently spread rapidly through populations of its newly acquired host [7], and has been found in all *An. anguilla* populations except those in Iceland [8]. *A. crassus* can thus be regarded as a model for the introduction and spread of invasive parasites [9].

In *An. anguilla*, both prevalence and mean intensity of infection by *A. crassus* are higher than in *An. japonica*[10,11]. In *An. anguilla* infections, the adult nematodes are larger, have an earlier onset of reproduction, a greater egg output [12] and induce increased pathology, including thickening and inflammation of the swim bladder wall [13]. It has been suggested that the life history modifications and changed virulence observed in *A. crassus* in the new host are due to an inadequate immune response in *An. anguilla*[14]. *An. japonica* is capable of killing histotropic larvae of the parasite after vaccination [15] or under high infection pressure [16], but this does not happen in *A. anguilla*.

The genus *Anguillicola* is placed in the nematode suborder Spirurina (clade III *sensu*[17]) [18,19]. The Spirurina are exclusively parasitic and include important human pathogens (the causative agents of filariasis and ascariasis) as well as prominent veterinary parasites. Molecular phylogenetic analyses place *Anguillicola* in a clade of spirurine nematodes (Spirurina B of [20]) that have a freshwater or marine intermediate host, but infect a wide range of carnivorous definitive hosts. Spirurina B is sister to the main Spirurina C, including the agents of filariasis and ascariasis, and thus *A. crassus* may be used as an outgroup taxon to understand the evolution of parasitic phenotypes in these species.

The differences in the biology of *A. crassus* in *An. japonica* (coevolved) and *An. anguilla* (recently captured) eel hosts is likely to result from differential interactions between host genetics and parasite genetics. While genetic differences between the host species are expected, it is not known what part, if any, genetic differentiation between the invading European and endemic Asian parasites plays. European *A. crassus* are less genetically variable than parasites taken from Asian hosts [21], reflecting the derived nature of the invading populations and the likely population bottlenecks this entailed. As part of a programme to understand the invasiveness of *A. crassus* in *An. anguilla*, we are investigating differences in gene expression and genetic distinction between invading European and endemic Asian *A. crassus* exposed to the two host species.

Recent advances in sequencing technology (often termed next generation sequencing) provide the opportunity for rapid and cost-effective generation of genome-scale data. The Roche 454 platform [22] is particularly suited to transcriptomics of previously unstudied species [23]. Here we describe the generation of a reference transcriptome for *A. crassus* based on Roche 454 data, and explore patterns of gene expression and diversity within the nematode.

## Methods

### Nematode samples, RNA extraction, cDNA synthesis and Sequencing

*A. crassus* from *An. japonica* were sampled from Kao-Ping river and an adjacent aquaculture in Taiwan as described in [16]. Nematodes from *An. anguilla* were sampled from Sniardwy Lake, Poland and from the Linkenheimer Altrhein, Germany. After determination of the sex of adult nematodes, they were stored in RNA-later (Quiagen, Hilden, Germany) until extraction of RNA. RNA was extracted from individual adult male and female nematodes and from a population of second stage larvae (L2) (Table 1). For host contamination screening a liver sample from an uninfected *An. japonica* was also processed. RNA was reverse transcribed and amplified into cDNA using the MINT-cDNA synthesis kit (Evrogen, Moscow, Russia). Emulsion PCR and library preparation were performed for each cDNA library according to the manufacturer’s protocols (Roche/454 Life Sciences), and sequenced on a Roche 454 Genome Sequencer FLX.

**Table 1:** Sampling, trimming and pre-assembly screening, library statistics

**Sequencing library**	**E1**	**E2**	**L2**	**M**	**T1**	**T2**	**total**
lifecycle stage	adult female	adult female	L2 larvae	adult male	adult female	adult female	
source population	Europe Rhine	Europe Poland	Europe Rhine	Asia cultured	Asia cultured	Asia wild	
geolocation	49.0262N;	53.751959N;	49.0262N;	22.6418N;	22.6418N;	22.5074N;	
	8.310556E	21.730957E	8.310556E	120.4440E	120.4440E	120.4220E	
raw reads	209325	111746	112718	106726	99482	116366	756363
low quality reads	92744	10903	15653	15484	7947	27683	170414
*A. crassus* rRNA reads	76403	11213	30654	31351	24929	7233	181783
eel-host mRNA reads	4835	3613	1220	1187	7475	11741	30071
eel-host rRNA reads	13112	69	1603	418	514	38	15754
Cercozoa reads (rRNA)	0	0	5286	0	0	0	5286
valid reads	22231	85948	58302	58286	58617	69671	353055
span of valid reads (in bases)	7167338	24046225	16661548	17424408	14443123	20749177	100491819
reads mapping (uniquely)	12023	65398	39690	36782	42529	55966	252388
reads mapping to	8359	61073	12917	31673	37306	50445	201773
*A. crassus* contigs							
reads mapping highCA	5883	48009	8475	18998	28970	41963	152298
contigs							
reads mapping to contigs	3595	34115	1602	10543	21413	22909	94177
with count > 32							

Raw sequencing reads are archived under study-accession number SRP010313 in the NCBI Sequence Read Archive (SRA; http://trace.ncbi.nlm.nih.gov/Traces/sra/?study=SRP010313) [24]. All samples were sequenced using the FLX Titanium chemistry, except for the Taiwanese female sample T1, which was sequenced using FLX standard chemistry, to generate between 99,000 and 209,000 raw reads per sample. For the L2 library, which had a larger number of non-*A. crassus*, non-*Anguilla* reads, we confirmed that these data were not laboratory contaminants by screening Roche 454 data produced on the same run in independent sequencing lanes.

### Trimming, quality control and assembly

Raw sequences were extracted in FASTA format (with the corresponding qualities files) using sffinfo (Roche/454) and screened for MINT adapter sequences using cross-match [25] (with parameters -minscore 20 -minmatch 10). Seqclean [26] was used to identify and remove poly-A-tails, low quality, low complexity and short (<100 base) sequences. All reads were compared to a set of screening databases using BLAST [27] (expect value cutoff E <1e-5, low complexity filtering turned off: -F F). The databases used were (a) a host sequence database comprising an assembly of the *An. japonica* Roche 454 data, a unpublished assembly of *An. anguilla* Sanger dideoxy sequenced expressed sequence tags (made available to us by Gordon Cramb, University of St Andrews) and transcripts from EeelBase [28], a publicly available transcriptome database for the European eel; (b) a database of ribosomal RNA (rRNA) sequences from eel species derived from our Roche 454 data and EMBL-Bank; and (c) a database of rRNA sequences identified in our *A. crassus* data by comparing the reads to known nematode rRNAs from EMBL-Bank. This last database notably also contained cobiont rRNA sequences. Reads with matches to one of these databases over more than 80% of their length and with greater than 95% identity were removed from the dataset. Screening and trimming information was written back into sff-format using sfffile (Roche 454). The filtered and trimmed data were assembled using the combined assembly approach [23]: Two assemblies were generated, one using Newbler v2.6 [22] (with parameters -cdna -urt), the other using Mira v3.2.1 [29] (with parameters–job=denovo,est,accurate,454). The resulting two assemblies were combined into one using Cap3 [30] at default settings and contigs were labeled by whether they derived from both assemblies (high confidence assembly; highCA), or one assembly only (lowCA; for a detailed analysis of the assembly categories see the supporting Methods file). The superset of highCA contigs, lowCA contigs and the remaining unassembled reads defines the set of tentatively unique genes (TUGs).

### Post-assembly classification and taxonomic assignment of contigs

We rescreened the assembly for host and other contamination by comparing it (using BLAST) to the three databases defined above, and also to NEMBASE4, a nematode transcriptome database derived from whole genome sequencing and EST assemblies [31,32]. For each contig, the highest-scoring match was recorded, if it spanned more than 50% of the contig. We also compared the contigs to the NCBI non-redundant nucleotide (NCBI-nt) and protein (NCBI-nr) databases, recording the taxonomy of all best matches with expect values better than 1e-05. Sequences with a best hit to non-Metazoans or to Chordata within Metazoa were excluded from further analysis.

### Protein prediction and annotation

Protein translations were predicted from the contigs using prot4EST (version 3.0b) [33]. Proteins were predicted either by joining single high scoring segment pairs (HSPs) from a BLAST search of uniref100 [34], or by ESTscan [35], using as training data the *Brugia malayi* complete proteome [36] back-translated using a codon usage table derived from the BLAST HSPs, or, if the first two methods failed, simply the longest ORF in the contig. For contigs where the protein prediction required insertion or deletion of bases in the original sequence, we also imputed an edited sequence for each affected contig. Annotations with Gene Ontology (GO), Enzyme Commission (EC) and Kyoto Encyclopedia of Genes and Genomes (KEGG) terms were inferred for these proteins using annot8r (version 1.1.1) [37], using the annotated sequences available in uniref100 [34]. Up to 10 annotations based on a BLAST similarity bitscore cut-off of 55 were obtained for each annotation set. The complete *B. malayi* proteome (as present in uniref100) and the complete *C. elegans* proteome (as present in WormBase v.220) were also annotated in the same way. SignalP V4.0 [38] was used to predict signal peptide cleavage sites and signal anchor signatures for the *A. crassus* transcriptome and for the proteomes of the two model nematodes. InterProScan [39] (command line utility iprscan version 4.6 with options -cli -format raw -iprlookup -seqtype p -goterms) was used to obtain domain annotations for the highCA contigs. We recorded the presence of a lethal RNAi phenotype in the *C. elegans* ortholog of each TUG using the biomart-interface [40] to WormBase v. 220 using the R package biomaRt [41].

### Single nucleotide polymorphism analysis

We mapped the raw reads to the complete set of contigs, replacing imputed sequences for originals where relevant, using ssaha2 (with parameters -kmer 13 -skip 3 -seeds 6 -score 100 -cmatch 10 -ckmer 6 -output sam -best 1) [42]. From the ssaha2 output, pileup files were produced using samtools [43], discarding reads mapping to multiple regions. VarScan [44] (pileup2snp) was used with default parameters on pileup files to output lists of single nucleotide polymorphisms (SNPs) and their locations.

In the 10,496 SNPs thus defined, the ratio of transitions (ti; 6,908) to transversion (tv; 3,588) was 1.93. From the prot4EST predictions, 7,189 of the SNPs were predicted to be inside an ORF, with 2,322 at codon first positions, 1,832 at second positions and 3,035 at third positions. As expected, ti/tv inside ORFs (2.39) was higher than outside ORFs (1.25). The ratio of synonymous polymorphisms per synonymous site to non-synonymous polymorphisms per non-synonymous site in this unfiltered SNP set (dn/ds) was 0.45, rather high compared to other analyses. Roche 454 sequences have well-known systematic errors associated with homopolymeric nucleotide sequences [45], and the effect of exclusion of SNPs in, or close to, homopolymer regions was explored. When SNPs were discarded using different size thresholds for homopolymer runs and proximity thresholds, the ti/tv and in dn/ds ratios changed (Additional file 1: Figure S1). Based on this SNPs associated with a homopolymer run longer than 3 bases within a window of 11 bases (5 bases to the right, 5 to the left) around the SNP were discarded. There was a relationship between TUG dn/ds and TUG coverage, associated with the presence of sites with low abundance minority alleles (less than 7% of the allele calls), suggesting that some of these may be errors. Removing low abundance minority allele SNPs from the set removed this effect (Additional file 1: Figure S2). For enrichment analysis of GO terms associated with positively selected TUGs we used the R package GOstats [46].

Using Samtools [43] (mpileup -u) and Vcftools [47] (view -gcv) we genotyped individual libraries for each of the master list of SNPs. Genotype- calls were accepted at a phred-scaled genotype quality threshold of 10. In addition to the relative heterozygosity (number of homozygous sites/number of heterozygous sites) we used the R package Rhh [48] to calculate internal relatedness [49], homozygosity by locus [50] and standardised heterozygosity [51] from these data. We confirmed the significance of heterozygote-heterozygote correlation by analysing the mean and 95% confidence intervals from 1000 bootstrap replicates estimated for all measurements.

### Gene expression analysis

Read-counts were obtained from the bam files generated for genotyping using the R package Rsamtoools [52]. LowCA contigs and contigs with fewer than 32 reads over all libraries were excluded from analysis. Libraries E1 and L2 had very low overall counts and thus we excluded these libraries from analysis. The statistic of Audic and Claverie [53] as implemented in ideg6 [54] was used to contrast single libraries. Differential expression between libraries from male versus female nematodes was accepted for genes that differed in expression values between all the female libraries (E2, T1 and T2; see Table 1) versus the male (M) library (p <0.01), but had no differential expression within any of the female libraries at the same threshold. Differential expression between libraries from nematodes of European *An. anguilla* and Taiwanese *An. japonica* origin was accepted for genes that differed in expression values between library E2 and both libraries T1 and T2 (p <0.01), but showed no differences between T1 and T2.

### Overrepresentation analyses

The R package annotationDbi [55] was used to obtain a full list of associations (along with higher-level terms) from annot8r annotations prior to analysis of GO term overrepresentation in gene sets selected on the basis of dn/ds or expression values. The R package topGO [56] was used to traverse the annotation graph and analyse each node term for overrepresentation in the focal gene set compared to an appropriate universal gene set (all contigs with dn/ds values or all contigs analysed for gene expression) with the “classic” method and Fisher’s exact test. Terms for which an offspring term was already in the table and no additional counts supported overrepresentation were removed. Mann-Whitney u-tests were used to test the influence of factors on dn/ds values. To investigate multiple contrasts between groups (factors) Nemenyi-Damico-Wolfe-Dunn tests were used, and for overrepresentation of one group (factor) in other groups (factors) Fisher’s exact test was used.

### General coding methods

The bulk of analysis (unless otherwise described) presented was carried out in R [57] using custom scripts. For visualisation we used the R packages ggplot2 [58] and VennDiagram [45].

## Results

### Sampling *A. crassus*

One female *A. crassus* and one male *A. crassus* were sampled from an *An. japonica* aquaculture with high infection loads in Taiwan, and an additional female was sampled from an *An. japonica* caught in a stream with low infection pressure adjacent to the aquaculture. A female nematode and pool of L2 were sampled from *An. anguilla* in the river Rhine, and one female from *A. anguilla* from a lake in Poland. All adult nematodes were replete with host blood. To assist in downstream filtering of host from nematode reads, we also sampled RNA from the liver of an uninfected Taiwanese *An. japonica*.

### Assembly and post-assembly screening

A total of 756,363 raw sequencing reads were generated for *A. crassus* (Table 1). These were rigorously filtered (see supporting infromation) and 353,055 remaining reads (spanning 100,491,819 bases) were assembled using the combined assembler strategy [23], employing Roche 454 gsAssembler (also known as Newbler; version 2.6) and MIRA (version 3.21) [29] (Additional file 2). This coassembly will be included in future versions of nembase (nembase5) and is available at http://www.anguillicola.nematod.es (further contig data can be found in Additional file 3). It comprised 13,851 contigs supported by both assembly algorithms, 3,745 contigs supported by only one of the assembly algorithms and 22,591 singletons that not assembled by either program (Table 2). Contigs supported by both assemblers were longer than those supported by only one assembler, and were more likely to have a significant similarity to previously sequenced protein coding genes than contigs assembled by only one of the algorithms, or the remaining unassembled singletons. These constitute the highCA, while those with evidence from only one assembler and the singletons are the lowCA. These datasets were the most parsimonious (having the smallest size) for their quality (covering the largest amount of sequence in reference transcriptomes). In the highCA parsimony and low redundancy was prioritised, while in the complete assembly (highCA plus lowCA including singletons) completeness was prioritised. The 40,187 sequences (contig consensuses and singletons) in the complete assembly are referred to as tentatively unique genes (TUGs).

**Table 2:** Assembly classification and contig statistics

	**highCA**	**lowCA**	**all TUGs**
total contigs	13851	26336	40187
contigs hitting rRNA	59	829	888
contigs hitting eel-mRNA or Chordata	1022	2419	3441
non-eukaryote contigs	1398	1935	3333
contigs remaining	11372	21153	32525
total span of remainingcontigs (in bases)	6575121	6157974	12733095
non-unique mean basecoverage of contigs	10.97923	14.66512	12.840
unique mean basecoverage of contigs	6.838352	2.443292	4.624
protein predictions byBLAST similarity	5664	4357	10021
protein predictions byESTscan	3597	8324	11921
protein predictions bylongest ORF	2085	8352	10437
contigs without proteinprediction	14	93	107
contigs with complete3’ end	2714	5909	8623
contig with complete5’ end	1270	1484	2754
full length contigs	185	104	289
contigs with GO-annotation	3875	2636	6511
contigs with EC-annotation	1493	967	2460
contigs with KEGG-annotation	2237	1609	3846
contigs with InerProScan- annotation	7557	n/d	
contigs with BLAST hit tonematode	5821	4869	10690
contigs with any BLAST hit	6008	5107	11115

We screened the complete assembly for remaining host contamination, and identified 3,441 TUGs that had significant, higher similarity to eel (and/or chordate; EMBLBank Chordata proteins) than to nematode sequences [32]. Given the identification of cercozoan ribosomal RNAs in the L2 library, we also screened the complete assembly for contamination with transcripts from other taxa.

1,153 TUGs were found with highest significant similarity to Eukaryota outside of the kingdoms Metazoa, Fungi and Viridiplantae. These contigs matched genes from a wide range of protists from Apicomplexa (mainly Sarcocystidae, 28 hits and Cryptosporidiidae 10 hits), Bacillariophyta (diatoms, mainly Phaeodactylaceae, 41 hits), Phaeophyceae (brown algae, mainly Ectocarpaceae, 180 hits), Stramenopiles (Albuginaceae, 63 hits), Kinetoplasitda (Trypanosomatidae, 26 hits) and Heterolobosea (Vahlkampfidae, 38 hits). Additionally 298 TUGs had best, significant matches to genes from fungi (e.g Ajellomycetaceae, 53 hits) and 585 TUGs had best, significant matches to genes from plants. Outside the Eukaryota there were significant best matches to Bacteria (825 TUGs; mostly to members of the Proteobacteria), Archaea (8 TUGs) and viruses (9 TUGs). No TUGs had significant, best matches to *Wolbachia* or related Bacteria known as symbionts of nematodes and arthropods. All TUGs with highest similarity to sequences deriving from taxa outside Metazoa were excluded. The final, screened *A. crassus* assembly has 32,525 TUGs, spanning 12,733,095 bases (of which 11,372 are highCA-derived, and span 6,575,121 bases). All analyses reported below are based on this filtered dataset.

### Annotation

**Figure 1:**
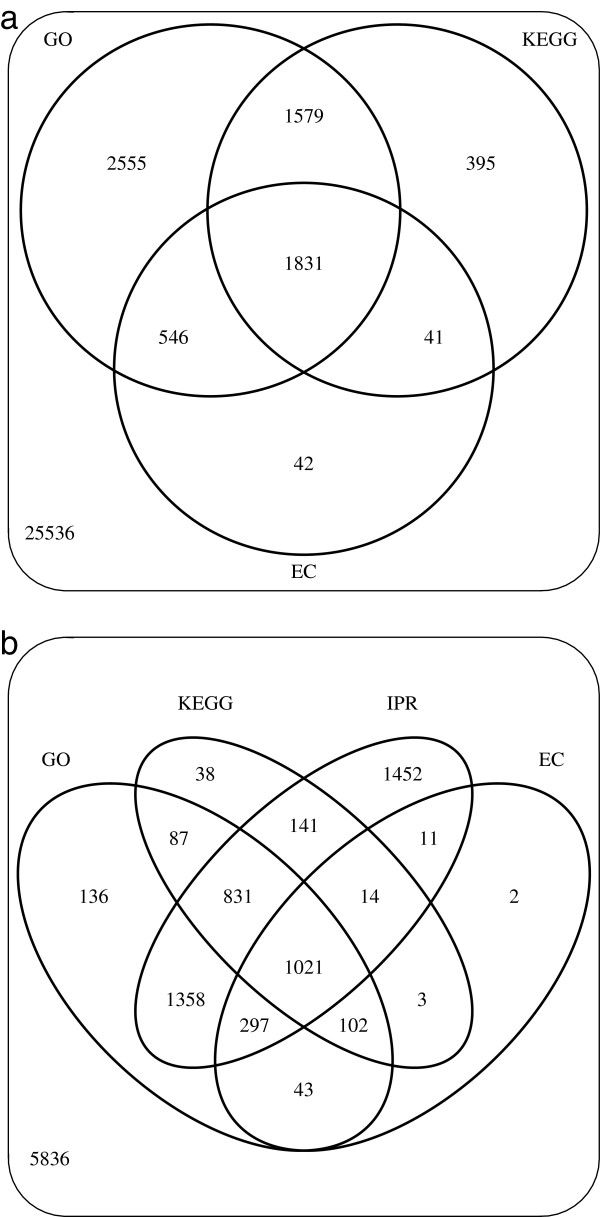
**Annotation of the Anguilicolla crassus transcriptome.** Number of annotated sequences in the transcriptome of *A. crssus* for all TUGs **(a)** and for highCA contigs **(b)**. Annotations with Gene Ontology (GO), Enzyme Commission (EC) and Kyoto Encyclopedia of Genes and Genomes (KEGG) terms were inferred for predicted proteins using annot8r (version 1.1.1) [37]. For highCA contigs additional domain annotations obtained with InterProScan [39] are also enumerated.

For 32,418 of the *A. crassus* TUGs a protein was predicted using prot4EST [33] (Table 2). An apparently full-length open reading frame (ORF) was identified in 353 TUGs, while for 29,877 the 5’ ends and for 24,277 the 3’ ends were missing. In 13,383 TUGs the corrected sequence with the imputed ORF was slightly changed compared to the raw sequence by insertions or deletions necessary to obtain a continuous reading frame. Using BLAST we determined that 9,556 had significant similarity to *C. elegans* proteins, 9,664 TUGs matched *B. malayi* proteins, and 11,620 TUGs had matches in NEMPEP4 [31,32]. Comparison to the UniProt reference identified 11,115 TUGs with significant similarities. We used annot8r [37] to assign GO terms to 6,511 TUGs, EC numbers for 2,460 TUGs and KEGG pathway annotations for 3,846 TUGs (Table 2). Additionally 5,125 highCA contigs were annotated with GO terms through InterProScan [39]. Nearly one third (6,989) of the *A. crassus* TUGs were annotated with at least one identifier, and 1,831 had GO, EC and KEGG annotations (Figure 1).

**Figure 2:**
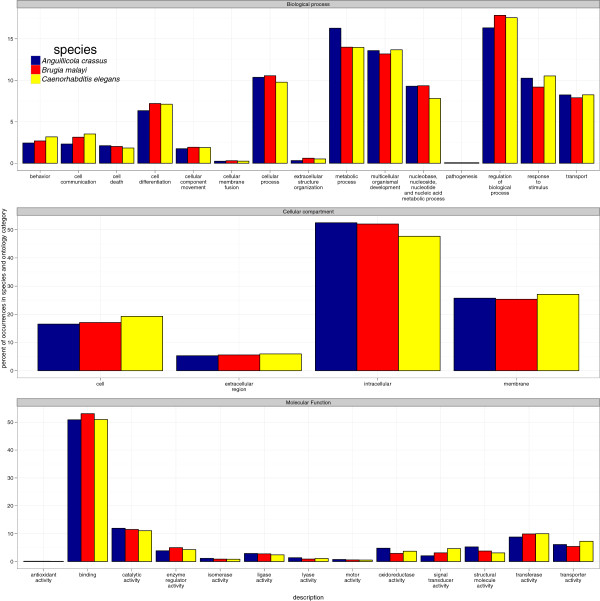
**Comparing high level GO-slim annotations.** Comparing high level GO-slim annotations obtained through annot8r (version 1.1.1) [37] for *A. crassus* to those for the model nematodes *C. elegans* and *B. malayi* infered using the same pipeline. For GO categories molecular function, cellular compartment and biological process the number of terms in high level GO-slim categories is given. In the two parasitic nematodes a higher degree of congruence in annotation spectrum is observed (Spearman correlation coefficient 0.95) than in comarison to the complete proteome of *C. elegans* (0.90).

We compared our *A. crassus* GO annotations for high-level GO-slim terms to the annotations (obtained in the same way) for the complete proteome of the spirurid filarial nematode *B. malayi* and the complete proteome of *C. elegans* (Figure 2). The occurrence of GO terms in the annotation of the partial transcriptome of *A. crassus* was more similar to that of the proteome of *B. malayi* (0.95; Spearman correlation coefficient) than to the that of the proteome of *C. elegans* (0.9).

Despite the lack of completeness at the 5’ end suggested by peptide prediction, just over 3% of the TUGs were predicted to be secreted (920 with signal peptide cleavage sites and 65 signal peptides with a transmembrane signature). Again these predictions are more similar to predictions using the same methods for the proteome of *B. malayi* (742 signal peptide cleavage sites and 41 with transmembrane anchor) than for the proteome of *C. elegans* (4,273 signal peptide cleavage sites and 154 with transmembrane anchor).

By comparison to RNAi phenotypes for *C. elegans* genes [59,60] likely to be orthologous to *A. crassus* TUGs, 6,029 TUGs were inferred to be essential (RNAi lethal phenotype in *C. elegans*).

To explore the phylogenetic conservation of *A. crassus* TUGs, they were classified as conserved across kingdoms, conserved in Metazoa, conserved in Nematoda, conserved in Spirurina or novel to *A. crassus* by comparing them to custom database subsets using BLAST (Table 3). Using a relatively strict cutoff, a quarter of the highCA contigs were conserved across kingdoms, and 10% were apparently restricted to Nematoda. Nearly half of the highCA contigs were novel to *A. crassus*.

**Table 3:** Evolutionary conservation and novelty

**contig set**	**cutoff**	**conserved**	**Metazoa**	**Nematoda**	**Clade3**	**Ac**
all TUGs	50	5604	1715	2173	1485	21548
all TUGs	80	3506	1383	2015	1525	24096
highCA	50	3479	876	1010	601	5406
highCA	80	2457	833	1084	716	6282

Similar patterns were observed for conservation assessed at different stringency, and when assessed across all TUGs, except that a higher proportion of all TUGs were apparently unique to *A. crassus*.

**Figure 3:**
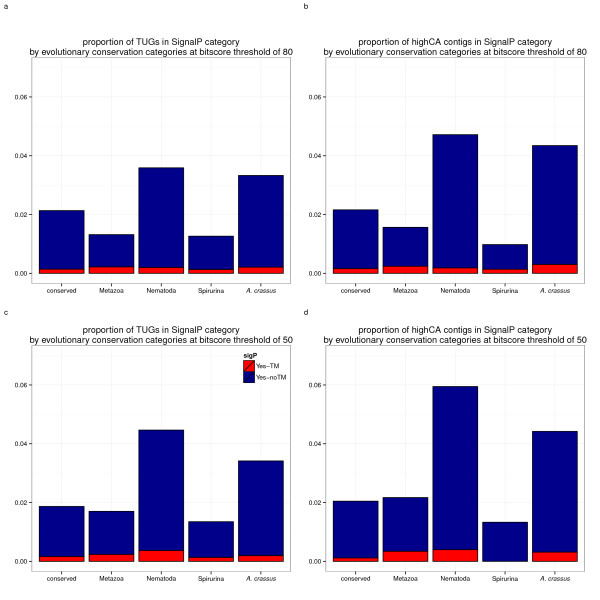
**Enrichment of Signal-positives for categories of evolutionary conservations.** Categories of evolutionary conservation recorded using the taxonomy of BLAST-matches at two different bitscore thresholds (50 or 80) are compared for the occurence of signal peptide cleavage sites and signal anchor signatures, predicted using SignalP V4.0 [38]. Contigs were categorised as conserved, novel in the kingdom Metazoa, the phylum Nematoda or Spirurina *sensu*[17]. TUGs without any match at a given threshold were categorised as novel in *A. crassus* (Ac). The highest proportions of genes predicted to have secretory signal peptides are observed in TUGs predicted to be part of gene families that arose in the last common ancestor of Nematoda or to be novel to *A. crassus*.

Proteins predicted to be restricted to Nematoda and novel in *A. crassus* were significantly enriched in signal peptide annotation compared to conserved proteins, proteins novel in Metazoa and novel in Spirurina (Fisher’s exact test p <0.001 ; Figure 3). The proportion of lethal RNAi phenotypes was significantly higher for *C. elegans* presumed orthologs of TUGs conserved across kingdoms (97.23%) than for orthologs of TUGs not conserved across kingdoms (94.59%; p <0.001, Fisher’s exact test).

### Identification and analysis of single nucleotide polymorphisms

Single nucleotide polymorphisms (SNPs) were called using VARScan [44] on the 1,100,522 bases of TUGs that had coverage of more than 8-fold available. SNPs predicted to have more than 2 alleles, or that mapped to an undetermined (N) base were excluded, as were SNP likely to be due to base calling errors close to homopolymer tracts and SNP calls resulting from apparent rare variants.

Our filtered SNP dataset includes 5,113 SNPs, with 4.65 SNPs per kb of contig sequence (Additional file 4). There were 7.95 synonymous SNPs per 1000 synonymous bases and 2.44 non-synonymous SNPs per 1000 non synonymous bases. A mean dn/ds of 0.244 was calculated for the 765 TUGs (all highCA contigs) containing at least one synonymous SNP. Positive selection can be inferred from high dn/ds ratios. Overrepresented GO ontology terms associated with TUGs with dn/ds higher than 0.5 were identified (Table 4; Additional file 5: Figure S11 a, b, c). Within the molecular function category, “peptidase activity” was the most significantly overrepresented term. Twelve of the thirteen high dn/ds TUGs annotated as peptidases each had unique orthologs in *C. elegans* and *B. malayi*. Other overrepresented categories identified subunits of the respiratory chain: “heme-copper terminal oxidase activity” and “cytochrome-c oxidase activity” in molecular function and “mitochondrion” in cellular compartment. Contigs identified as novel to Spirurina and novel in *A. crassus* had a significantly higher dn/ds than other contigs (Additional file 1: Figure S3).

**Table 4:** **Overrepresentation of GO terms in positively selected *A. crassus *TUGs**

**Category**	**GO.ID**	**Term**	**Number annotated**	**Number dn/ds > 0.5**	**Expected**	**p.value**
molecular function	GO:0008233	peptidase activity	43	13	6.08	0.0034
	GO:0015179	L-amino acid transmembrane transporter activity	2	2	0.28	0.0198
	GO:0043021	ribonucleoprotein complex binding	6	3	0.85	0.0396
	GO:0070011	peptidase activity, acting on L-amino acid peptides	35	9	4.95	0.0442
	GO:0004175	endopeptidase activity	25	7	3.54	0.0488
biological process	GO:0042594	response to starvation	15	7	2.13	0.0022
	GO:0009083	branched chain family amino acid catabolic process	3	3	0.43	0.0027
	GO:0006914	autophagy	12	6	1.70	0.0031
	GO:0009063	cellular amino acid catabolic process	10	5	1.42	0.0071
	GO:0009267	cellular response to starvation	7	4	0.99	0.0093
	GO:0006520	cellular amino acid metabolic process	44	12	6.24	0.0128
	GO:0006915	apoptotic process	78	18	11.06	0.0147
	GO:0009308	amine metabolic process	57	14	8.08	0.0189
	GO:0005997	xylulose metabolic process	2	2	0.28	0.0199
	GO:0006739	NADP metabolic process	2	2	0.28	0.0199
	GO:0007616	long-term memory	2	2	0.28	0.0199
	GO:0009744	response to sucrose stimulus	2	2	0.28	0.0199
	GO:0010172	embryonic body morphogenesis	2	2	0.28	0.0199
	GO:0015807	L-amino acid transport	2	2	0.28	0.0199
	GO:0050885	neuromuscular process controlling balance	2	2	0.28	0.0199
	GO:0007281	germ cell development	17	6	2.41	0.0226
	GO:0090068	positive regulation of cell cycle process	17	6	2.41	0.0226
	GO:0042981	regulation of apoptotic process	64	15	9.07	0.0232
	GO:0051329	interphase of mitotic cell cycle	23	7	3.26	0.0320
	GO:0044106	cellular amine metabolic process	55	13	7.80	0.0325
	GO:0031571	mitotic cell cycle G1/S transition DNA damage checkpoint	14	5	1.98	0.0355
	GO:0010564	regulation of cell cycle process	34	9	4.82	0.0377
	GO:0006401	RNA catabolic process	6	3	0.85	0.0398
	GO:0010638	positive regulation of organelle organization	6	3	0.85	0.0398
	GO:0009056	catabolic process	149	28	21.12	0.0398
	GO:0008219	cell death	93	19	13.18	0.0441
	GO:0007154	cell communication	144	27	20.41	0.0455
	GO:0051726	regulation of cell cycle	52	12	7.37	0.0474
	GO:0030330	DNA damage response, signal transduction by p53 class mediator	15	5	2.13	0.0475
	GO:0033238	regulation of cellular amine metabolic process	15	5	2.13	0.0475
cellular compartment	GO:0030532	small nuclear ribonucleoprotein complex	7	4	0.99	0.0093
	GO:0005739	mitochondrion	137	28	19.38	0.0113
	GO:0005682	U5 snRNP	2	2	0.28	0.0198
	GO:0015030	Cajal body	2	2	0.28	0.0198
	GO:0046540	U4/U6 x U5 tri-snRNP complex	2	2	0.28	0.0198
	GO:0016607	nuclear speck	6	3	0.85	0.0396

Signal peptide containing proteins have been shown to have higher rates of evolution than cytosolic proteins in a number of nematode species. *A. crassus* TUGs predicted to contain signal peptide cleavage sites showed a non-significant trend towards higher dn/ds values than TUGs without signal peptide cleavage sites (p=0.22; two sided Mann-Whitney-test). Orthologs of *C. elegans* transcripts with lethal RNAi phenotype are expected to evolve under stronger selective constraints and the values of dn/ds showed a non-significant trend towards lower values in TUGs with orthologs with a lethal phenotype compared to a non-lethal phenotypes (p=0.815, two-sided U-test).

The genotypes of single adult nematodes were called using Samtools [43] and Vcftools [47], and 199 informative sites (where two alleles were found in at least one assured genotype at least in one of the nematodes) were identified in 152 contigs. Internal relatedness [49], homozygosity by loci [50] and standardised heterozygosity [51] all identified the Taiwanese nematode from aquaculture (sample T1) as the most heterozygous and the European nematode from Poland (sample E2) as the least heterozygous individuals (Table 5).

**Table 5:** Measurements of multi-locus heterozygosity for single worms

	**Relative heterozygosity**	**Internal relatedness**	**Homozygosity by loci**	**Standardised heterozygosity**	**Informative SNPs**
T2	0.45	-0.73	0.59	1.00	121
T1	0.93	-0.95	0.34	1.62	136
M	0.37	-0.73	0.66	0.84	92
E1	0.38	-0.83	0.60	0.91	65
E2	0.18	-0.35	0.82	0.50	140

The genome-wide representativeness of these 199 SNP markers for the whole genome in population genetic studies was confirmed using heterozygosity-heterozygosity correlation [48]: mean internal relatedness = 0.78, lower bound of 95% confidence intervals from 1000 bootstrap replicates (cil) = 0.444; mean homozygosity by loci = 0.86, cil = 0.596; standardised heterozygosity = 0.87, cil= 0.632.

### Differential gene expression

Gene expression was inferred by the unique mapping of 252,388 (71.49%) of the raw reads to the fullest assembly (including all assembled contigs as a “filter”; total contigs/all TUGs in Table 2). Non-*A. crassus* contigs, and all contigs with fewer than 32 reads overall were excluded. Thus 658 TUGs were analysed for differential expression using ideg6 for normalisation and the statistic of Audic and Claverie [53] for detection of differences. Of these TUGs, 54 showed expression predominantly in the male library, 56 TUGs were more highly represented in the female library (Additional file 1), 56 TUGs were primarily expressed in the libraries from Taiwan, and 22 TUGs were overrepresented in the European library (Additional file 6).

Analysis of overrepresentation of of GO terms associated with TUGs differentially expressed between male and female libraries identified ribosomal proteins, oxidoreductases and collagen processing enzyme terms (Table 6; Additional file 5: Figure S11 g, h, i). The ribosomal proteins were all overexpressed in the male library, while the oxidoreductases and collagen processing enzymes were overexpressed in female libraries. Similar analysis of overrepresentation of of GO terms associated with the TUGs differentially expressed between European nematodes and Asian nematodes identified several terms of catalytic activity related to metabolism (Table 7; Additional file 5: Figure S11 d, e, f).

**Table 6:** **Overrepresentation of GO-terms differentially expressed between male and female *A. crassus***

**Category**	**GO.ID**	**Term**	**Number annotated**	**Number significant**	**Expected**	**p.value**
molecular function	GO:0005198	structural molecule activity	52	18	8.39	0.00024
	GO:0016706	oxidoreductase activity, acting on paired donors, with incorporation or reduction of molecular oxyge...	3	3	0.48	0.00400
	GO:0004656	procollagen-proline 4-dioxygenase activity	2	2	0.32	0.02562
biological process	GO:0034621	cellular macromolecular complex subunit organization	73	22	11.42	0.00024
	GO:0034641	cellular nitrogen compound metabolic process	161	37	25.19	0.00024
	GO:0048731	system development	150	35	23.47	0.00035
	GO:0071822	protein complex subunit organization	71	21	11.11	0.00050
	GO:0043933	macromolecular complex subunit organization	82	23	12.83	0.00057
	GO:0032774	RNA biosynthetic process	72	21	11.26	0.00063
	GO:0000022	mitotic spindle elongation	20	9	3.13	0.00122
	GO:0006139	nucleobase-containing compound metabolic process	141	32	22.06	0.00189
	GO:0071841	cellular component organization or biogenesis at cellular level	140	31	21.90	0.00418
	GO:0050789	regulation of biological process	201	40	31.44	0.00474
	GO:0071842	cellular component organization at cellular level	136	30	21.28	0.00575
	GO:0090304	nucleic acid metabolic process	107	25	16.74	0.00673
	GO:0040007	growth	139	30	21.75	0.00867
	GO:0016070	RNA metabolic process	98	23	15.33	0.00988
	GO:0007275	multicellular organismal development	222	42	34.73	0.01108
	GO:0009791	post-embryonic development	117	26	18.30	0.01201
	GO:0034976	response to endoplasmic reticulum stress	7	4	1.10	0.01306
	GO:0042157	lipoprotein metabolic process	7	4	1.10	0.01306
	GO:0040010	positive regulation of growth rate	62	16	9.70	0.01505
	GO:0018996	molting cycle, collagen andcuticulin-based cuticle	23	8	3.60	0.01557
	GO:0042274	ribosomal small subunit biogenesis	11	5	1.72	0.01706
	GO:0048856	anatomical structure development	219	41	34.26	0.01880
	GO:0022414	reproductive process	109	24	17.05	0.02003
	GO:0032501	multicellular organismal process	242	44	37.86	0.02062
	GO:0065007	biological regulation	220	41	34.42	0.02114
	GO:0071840	cellular component organization or biogenesis	172	34	26.91	0.02135
	GO:0009416	response to light stimulus	8	4	1.25	0.02310
	GO:0008543	fibroblast growth factor receptor signaling pathway	2	2	0.31	0.02407
	GO:0018401	peptidyl-proline hydroxylation to4-hydroxy-L-proline	2	2	0.31	0.02407
	GO:0046887	positive regulation of hormonesecretion	2	2	0.31	0.02407
	GO:0071570	cement gland development	2	2	0.31	0.02407
	GO:0016043	cellular component organization	168	33	26.28	0.02835
	GO:0009792	embryo development ending in birth or egg hatching	124	26	19.40	0.02873
	GO:0009152	purine ribonucleotide biosynthetic process	5	3	0.78	0.02876
	GO:0000279	M phase	45	12	7.04	0.02921
	GO:0002164	larval development	107	23	16.74	0.03246
	GO:0070727	cellular macromolecule localization	31	9	4.85	0.03530
	GO:0042254	ribosome biogenesis	22	7	3.44	0.03929
	GO:0048518	positive regulation of biological process	127	26	19.87	0.04015
	GO:0022613	ribonucleoprotein complex biogenesis	27	8	4.22	0.04202
	GO:0007010	cytoskeleton organization	58	14	9.07	0.04305
	GO:0000003	reproduction	141	28	22.06	0.04750
	GO:0044267	cellular protein metabolic process	135	27	21.12	0.04864
cellular compartment	GO:0005634	nucleus	163	38	25.71	0.00010
	GO:0030529	ribonucleoprotein complex	64	20	10.09	0.00034
	GO:0043232	intracellular non-membrane-bounded organelle	116	28	18.30	0.00187
	GO:0044444	cytoplasmic part	260	48	41.01	0.00194
	GO:0043231	intracellular membrane-boundedorganelle	253	47	39.91	0.00294
	GO:0005829	cytosol	151	33	23.82	0.00359
	GO:0031981	nuclear lumen	68	18	10.73	0.00725
	GO:0005618	cell wall	18	7	2.84	0.01279
	GO:0043229	intracellular organelle	272	48	42.90	0.01372
	GO:0070013	intracellular organelle lumen	94	22	14.83	0.01377
	GO:0044446	intracellular organelle part	195	38	30.76	0.01470
	GO:0009536	plastid	28	9	4.42	0.01871
	GO:0045169	fusome	2	2	0.32	0.02446
	GO:0070732	spindle envelope	2	2	0.32	0.02446
	GO:0022627	cytosolic small ribosomal subunit	16	6	2.52	0.02606
	GO:0005791	rough endoplasmic reticulum	5	3	0.79	0.02939
	GO:0009507	chloroplast	26	8	4.10	0.03508
	GO:0005773	vacuole	46	12	7.26	0.03660
	GO:0005811	lipid particle	31	9	4.89	0.03690

**Table 7:** **Overrepresentation of GO-terms differentially expressed between Taiwanese and European *A. crassus***

**Category**	**GO.ID**	**Term**	**Number annotated**	**Number significant**	**Expected**	**p.value**
molecular function	GO:0016453	C-acetyltransferase activity	3	3	0.37	0.0018
	GO:0003824	catalytic activity	158	27	19.50	0.0079
	GO:0016746	transferase activity, transferring acylgroups	8	4	0.99	0.0097
	GO:0003682	chromatin binding	2	2	0.25	0.0149
	GO:0003985	acetyl-CoA C-acetyltransferase activity	2	2	0.25	0.0149
	GO:0008061	chitin binding	2	2	0.25	0.0149
	GO:0003713	transcription coactivator activity	6	3	0.74	0.0268
	GO:0005543	phospholipid binding	6	3	0.74	0.0268
	GO:0004090	carbonyl reductase (NADPH) activity	3	2	0.37	0.0412
	GO:0008289	lipid binding	12	4	1.48	0.0473
	GO:0016853	isomerase activity	12	4	1.48	0.0473
biological process	GO:0016126	sterol biosynthetic process	5	4	0.60	0.00081
	GO:0044281	small molecule metabolic process	107	22	12.80	0.00105
	GO:0048732	gland development	9	5	1.08	0.00169
	GO:0006694	steroid biosynthetic process	10	5	1.20	0.00307
	GO:0006338	chromatin remodeling	4	3	0.48	0.00586
	GO:0006695	cholesterol biosynthetic process	4	3	0.48	0.00586
	GO:0042180	cellular ketone metabolic process	57	13	6.82	0.00800
	GO:0023051	regulation of signaling	29	8	3.47	0.01318
	GO:0001822	kidney development	2	2	0.24	0.01399
	GO:0006611	protein export from nucleus	2	2	0.24	0.01399
	GO:0009953	dorsal/ventral pattern formation	2	2	0.24	0.01399
	GO:0048581	negative regulation of post-embryonic development	2	2	0.24	0.01399
	GO:0051124	synaptic growth at neuromuscular junction	2	2	0.24	0.01399
	GO:0070050	neuron homeostasis	2	2	0.24	0.01399
	GO:0019752	carboxylic acid metabolic process	54	12	6.46	0.01417
	GO:0008152	metabolic process	268	37	32.06	0.01595
	GO:0019219	regulation of nucleobase-containing compound metabolic process	42	10	5.02	0.01617
	GO:0006355	regulation of transcription,DNA-dependent	30	8	3.59	0.01637
	GO:0010033	response to organic substance	62	13	7.42	0.01729
	GO:0019953	sexual reproduction	44	10	5.26	0.02265
	GO:0048747	muscle fiber development	6	3	0.72	0.02461
	GO:0032787	monocarboxylic acid metabolic process	21	6	2.51	0.02763
	GO:0051171	regulation of nitrogen compound metabolic process	52	11	6.22	0.02827
	GO:0048545	response to steroid hormonestimulus	16	5	1.91	0.03065
	GO:0048609	multicellular organismal reproductive process	60	12	7.18	0.03331
	GO:0009966	regulation of signal transduction	22	6	2.63	0.03462
	GO:0031325	positive regulation of cellularmetabolic process	28	7	3.35	0.03565
	GO:0009308	amine metabolic process	41	9	4.90	0.03874
	GO:0002026	regulation of the force of heart contraction	3	2	0.36	0.03877
	GO:0007595	lactation	3	2	0.36	0.03877
	GO:0030518	intracellular steroid hormonereceptor signaling pathway	3	2	0.36	0.03877
	GO:0034612	response to tumor necrosis factor	3	2	0.36	0.03877
	GO:0035071	salivary gland cell autophagic cell death	3	2	0.36	0.03877
	GO:0035220	wing disc development	3	2	0.36	0.03877
	GO:0043628	ncRNA 3’-end processing	3	2	0.36	0.03877
	GO:0045540	regulation of cholesterol biosynthetic process	3	2	0.36	0.03877
	GO:0051091	positive regulation of sequence-specific DNA binding transcription factor activity	3	2	0.36	0.03877
	GO:0051289	protein homotetramerization	3	2	0.36	0.03877
	GO:0002165	instar larval or pupal development	7	3	0.84	0.03951
	GO:0007589	body fluid secretion	7	3	0.84	0.03951
	GO:0048872	homeostasis of number of cells	7	3	0.84	0.03951
	GO:0060047	heart contraction	7	3	0.84	0.03951
	GO:0065008	regulation of biological quality	83	15	9.93	0.04013
	GO:0006066	alcohol metabolic process	35	8	4.19	0.04124
	GO:0050794	regulation of cellular process	154	24	18.42	0.04125
	GO:0006357	regulation of transcription fromRNA polymerase II promoter	12	4	1.44	0.04276
	GO:0006351	transcription, DNA-dependent	42	9	5.02	0.04489
	GO:0007276	gamete generation	42	9	5.02	0.04489
	GO:0005975	carbohydrate metabolic process	36	8	4.31	0.04827
cellular compartment	GO:0031967	organelle envelope	47	12	5.49	0.0031
	GO:0005740	mitochondrial envelope	29	8	3.38	0.0112
	GO:0005643	nuclear pore	2	2	0.23	0.0133
	GO:0005739	mitochondrion	93	17	10.85	0.0173
	GO:0031966	mitochondrial membrane	28	7	3.27	0.0312
	GO:0005902	microvillus	3	2	0.35	0.0369

TUGs annotated as acyltransferase were upregulated in the European libraries. However, the expression patterns for other TUGs with overrepresented terms connected to metabolism did not show concerted up or down-regulation. Thus for the term “steroid biosynthetic process”, 2 TUGs were downregulated and 3 contigs upregulated in European nematodes. No enrichment of signal peptide positive TUGs, of TUG conservation categories, or TUGs with *C. elegans* orthologs with lethal or non-lethal RNAi-phenotypes was identified. Significantly elevated dn/ds was found for TUGs differentially expressed in European versus Asian nematodes (Fisher’s exact test p=0.007; also both up- or down-regulated were significant). TUGs overexpressed in the female libraries showed elevated levels of dn/ds (Fisher’s exact test p=0.041), but contrast male overexpressed genes showed decreased levels of dn/ds (Fisher’s exact test p=0.014).

## Discussion

We have generated a *de novo* transcriptome for *A. crassus*, an important invasive parasite that threatens wild stocks of the European eel *An. anguilla*. These data will enable a broad spectrum of molecular research on this ecologically important and evolutionarily interesting parasite.

As *A. crassus* lives in close association with its host, we used exhaustive filtering to remove all host-derived, and host-associated organism-derived contamination from the raw and assembled data. We generated a transcriptome dataset from the definitive host *An. japonica* as part of this filtering process. In addition to eel-derived transcripts, we also removed data apparently derived from protists, particularly cercozoans, that may have been co-parasites of the eels sampled. Similar taxonomic screening of transcrioptome data has been shown to be important previously [61], particularly in rejection of hypotheses of horizontal gene transfer into the focal species [62]. We were not able to use base frequency and codon usage based screening to identify contaminant data [63,64] because contaminant sequences in our data derived from multiple genomes.

We used a combined assembly approach [23] to generate a transcriptome estimate that had low redundancy and high completeness. Projects using single assemblers often report substantially greater numbers of contigs for datasets of similar size (see e.g. [65]). The 3’ bias in the assembly likely derivesd from the use of oligod(T) in mRNA capture and cDNA synthesis and is near-ubiquitous in deep transcriptome sequencing projects (e.g. [66]). The final *A. crassus* TUG assembly (32,418 contig consensuses) spans 12.7 Mb, and thus likely covers most of the expected span of the transcriptome (the *C. elegans* transcriptome spans 30 Mb [67], and the *B. malayi* transcriptome 14 Mb [36]), albeit fragmented.

Comparison between free-living and parasitic nematode species can be used to identify genes that may underpin adaptations for parastism [68,69]. Annotations were derived for a 30% of all TUGs, and over 50% of the highCA contigs using sequence similarity to known proteins. Domain annotations were derived for 45% of the highCA TUGs using InterProScan [39]. Comparison with the complete proteomes of *B. malayi* and *C. elegans* showed a remarkable degree of congruence in annotation spectrum in the two parasitic nematodes. This implies that the *A. crassus* transcriptome is a representative partial genome [70]. Using a taxonomically-stratified analysis of BLAST similarities, we identified more *A. crassus* TUGs that apparently arose in the common ancestor of Nematoda than arose in the last common ancestor of the Spirurina. As *A. crassus* is part of a lineage that arises basally in Spirurina, the lack of genes associated with Sprirurina may be due to phylogenetic distance obscuring relationships, particularly if the genes underpinning parasitism are, as would be expected, rapidly evolving. TUGs predicted to be part of gene families that arose in the last common ancestor of Nematoda or to be novel to *A. crassus* contained the highest proportion of genes predicted to have secretory signal peptides. This confirms observations made in a *Nippostrongylus brasiliensis*[71], where secreted and surface proteins were less conserved. Analysis of dn/ds (see below) across conservation categories favors the hypothesis of rapid evolution in proteins with more restricted phylogenetic origins.

Transcriptome data were generated from multiple individual *A. crassus* of Taiwanese and European origin. We identified abundant SNPs both within and between populations, but noted aberrant patterns in the ratio of transitions to transversions (ti/tv) and the ratio of non-synonymous SNPs per non-synonymous site to synonymous SNPs per synonymous site (dn/ds). Screening of SNPs in or adjacent to homopolymer regions, removing “noise” associated with common homopolymer errors [72], improved overall measurements of SNP quality, increased the ti/tv ratio to more closely resemble that of canonical datasets, and resulted in a reduced, credible dn/ds ratio distribution. The corrected ti/tv value of 1.93 (1.25 outside and 2.39 inside ORFs) is in good agreement with the overall ti/tv of *Homo sapiens* (2.16 [73]) or *Drosophila melanogaster* (2.07 [74]). The mean dn/ds ratio decreased with removal of SNPs adjacent to homopolymer regions from 0.45 to 0.24. While interpretation of dn/ds ratios within populations is not unproblematic [75], the assumption of negative (purifying) selection on most protein coding genes makes lower mean values seem more plausible.

We applied a threshold value for the minority allele of 7% for exclusion of SNPs, as approximately 10 haploid equivalents were sampled (5 individual nematodes plus negligible contributions from the L2 library and offspring within the adult female nematodes). This screening reduced the number of non-synonymous SNPs in high coverage TUGs, removed the dependence of dn/ds on coverage, and removed the need to control for sampling biased by depth (i.e. coverage; see [76,77]).

The final dn/ds estimates seem plausible, as *D. melanogaster* female reproductive tract transcripts have dn/ds of 0.15 [78] and a Roche 454 transcriptomic analysis of the parasitic nematode *Ancylostoma caninum* reported dn/ds of 0.3 [79]. A dn/ds threshold (on coding sequence) of 0.5 has been suggested as threshold for assuming positive selection [78]. Using this we identified 144 TUGs that may be under positive selection, thirteen peptidases were under positive selection (out of 43 annotated), and the GO term peptidases was significantly overrepresented in the set of positively selected TUGs. Those thirteen peptidases are deeply conserved, as twelve had unique orthologue pairs in *B. malayi* and *C. elgans*. Peptidases have previously been proposed to have acquired prominent roles in host-parasite interactions. An *A. crassus* trypsin-like proteinase may be utilised by the tissue-dwelling third stage larvae to penetrate host tissue and an aspartyl proteinase may be a blood meal digestive enzyme in adults [2]. The thirteen proteinases under positive selection could be targets of adaptive immunity developed against *A. crassus*[15,80], which is often only elicited against some but not all larvae [81].

A set of 199 high-credibility SNPs with high information content for population genetic studies was identified by genotyping individual nematodes. The low number of SNPs inferred reflects both the variance in allele contribution introduced in transcriptomic data and the stringency of the software used, which is targeted at higher throughput genome sequence data [82]. Nevertheless, levels of genome-wide heterozygosity found for the five adult nematodes examined are in agreement with existing microsattelite data that show reduced heterozygosity in European populations of *A. crassus*[21]. The Polish female nematode was the most highly inbred, while the nematode from the cultured *An. japonica* from Taiwan was the most highly outbred.

While our experiment was not designed to identify differential expression between conditions (due to low replication) we used methods developed for comparison of cDNA libraries [53] to infer differential gene expression according to the origin of the sequencing libraries. This approach is widely used with 454 transcriptome data (e.g. [79]). We can only tentatively infer differential expression of a gene under different conditions (sex, origin) based on identification of significantly differential expression between libraries. Genes over-expressed in the male *A. crassus* included major sperm proteins [83], and, surprisingly, a suite of ribosomal proteins. Collagen processing enzymes were overexpressed in the female nematodes in line with modulation of collagen synthesis in nematode embryonic development, and the ovoviviparity of this species [83]. Acetyl-CoA acetyltransferase was identified as overexpressed in European nematodes compared to the Asian one. Acetyl-CoA acetyltransferases act in fatty-acid-oxidation in peroxisomes and mitochondria [84]. Together with a change in steroid metabolism and the enrichment of mitochondrially localised enzymes these suggest changes in the energy metabolism of *A. crassus* from different origins. Possible explanations could include a change to more or less aerobic processes in nematodes in Europe due to their bigger size and/or increased availability of nutrients. TUGs overexpressed in the female libraries showed elevated levels of dn/ds but genes overexpressed in males had decreased levels of dn/ds. The first finding is unexpected, as genes overexpressed in female libraries will also include TUGs related to larval development (such as the collagen modifying enzymes discussed above), and these larval transcripts in turn are expected to be under purifying selection because of pleiotropic effects of genes in early development [85]. The second contrasts with findings that male specific traits and transcripts often show hallmarks of positive selection [86,87]. In *A. caninum*, female-specific transcripts showed an enrichment of parasitism genes” [79] and a possible explanation would be a similar enrichment of positively selected parasitism-related genes in our dataset. For males the decreased dn/ds may be explained by the high number of ribosomal protein-encoding TUGs, which all show very low levels of dn/ds. That these TUGs were found to be differentially expressed remains puzzling. Some male-overexpressed TUGs, such as that encoding major sperm protein, showed elevated dn/ds. It is unlikely that correlation of differential expression with positive selection results from mapping artifacts, as all the ribosomal protein encoding TUGs identified overexpressed in males have very low dn/ds.

Genes differentially expressed according to the geographic origin of the nematodes showed significantly elevated levels of dn/ds. We interpret this as reflecting a correlation between sequence evolution and phenotypic modification in different host, environments or correlation between sequence evolution and evolution of gene expression. Whether expression of these genes is modified in different hosts or evolved rapidly in the contemporary divergence between European and Asian populations of *A. crassus*, is one focus of ongoing work building on the reference transcriptome presented here. For such an analysis it will be important to disentangle the influence of the host and the nematode population in common garden, co-inoculation experiments.

## Conclusions

The *A. crassus* transcriptome provides a basis for a new era of molecular research on this ecologically important species. It will aid not only analysis of the invasive biology of this parasite, assisting in identifying the origins of invading populations as well as the adaptations that may be selected in the new European host, but also in the investigation of the acquisition of parasitism in the great clade of animal parasites, Spirurina. In particular, positive selection of proteinases and differences in energy metabolism between European and Asian *A. crassus* constitute a candidate phenotype relevant for phenotypic modification or contemporary divergent evolution as well as for the long term evolution of parasitism.

## Competing interests

The authors declare that they have no competing interests.

## Authors’ contributions

EH, HT and MB conceived and designed the experiments. EH carried out bioinformatic analyses. SB assisted in bioinformatic analyses. AM prepared sequencing libraries. HT provided close supervision throughout. EH and MB interpreted results and prepared the manuscript. All authors have read and approved the final manuscript.

## Supplementary Material

Additional file 1 **A.crassus_transcriptome_sexDE.csv.** Contigs differentially expressed between male and female worms. Normalised counts and the natural logarithm of fold changes are given. Click here for file

Additional file 2 **Additional text. Supporting_information.pdf.** This document contains the 3 additional figures referenced in the main text and an additional text describing the assembly process and evaluation of assembly quality in further detail. This text contains additional 7 figures (additional figures 4-10) and 3 tables. Click here for file

Additional file 3 **Additional data. A.crassus_transcriptome_contig_data.csv.** All data computed on the contig level, as described in the manuscript and additional text including sequences (raw, coding, imputed, protein). Click here for file

Additional file 4 **A.crassus_transcriptome_screened_SNPs.csv.** High quality SNPs. The contig, the base relative to the start of the contig (base), the reference base-call (from), the alternative base-call (to), the number of reads supporting the reference (nfrom) and the alternative (nto), the percentage of the alternate allele (perc), whether the SNP is in the region of an ORF (inORF), the position in the Frame (inFrame) and the effect of the SNP (effect; synonymous, non-synonymous or nonsense) are given. Click here for file

Additional file 5 **Additional figure 11 (a-i). Subgraphs of GO induced by the top 10 terms identified as enriched in different sets of genes.** Subgraphs of the GO ontology categories induced by the top 10 terms identified as enriched in different sets of genes. Boxes indicate the 10 most significant terms. Box colour represents the relative significance, ranging from dark red (most significant) to light yellow (least significant). In each node the category-identifier, a (eventually truncated) description of the term, the significance for enrichment and the number of DE / total number of annotated gene is given. Black arrows indicate an “is-a” relationship. GO ontology category and the set of genes analysed for the enrichment are indicated in each figure. Click here for file

Additional file 6 **A.crassus_transcriptome_originDE.csv.** Contigs differentially expressed between European and Taiwanese worms. Normalised counts and the natural logarithm of fold changes are given. Click here for file
