# Supplementary material for: The transcriptome of the invasive eel swimbladder nematode parasite Anguillicola crassus
Source: BMC Genomics. 2013 Feb 8;14:87. doi: 10.1186/1471-2164-14-87 (PMC3630068; doi:10.1186/1471-2164-14-87)
Supplement: Additional file 1 — A.crassus_transcriptome_sexDE.csv. Contigs differentially expressed between male and female worms. Normalised counts and the natural logarithm of fold changes are given. [file 1471-2164-14-87-S1.pdf]

Addtional figures, tables and text describing the assembly process for "The transcriptome of the invasive eel swimbladder nematode parasite *Anguillicola crassus*" (Heitlinger et al.)

Contents

1 Additional figures referenced in the main text **2**

2 The transcriptome of *A.crassus* **6**

2.1 Sequencing, trimming and pre-assembly screening 6

3 Evaluating a method of combining assemblies **6**

3.1 Overview 6
3.2 The Newbler first-order assembly 6
3.3 The Mira assembly and the second-order assembly 8
3.4 Data categories in the second-order assembly 10
3.5 Contribution of first-order assemblies to second-order contigs . 11
3.6 Evaluation of the assemblies 13
3.7 Measurments on second-order assembly 17
3.7.1 Contig coverage 18
3.7.2 Example use of the contig-measurements 18
3.8 Finalising the fullest assembly set 20
3.9 Scrutinising the GO annotation 21

List of Figures

1 Filtering of putative SNPs 3
2 SNP distribution is affected by codon position and coverage . 4

3 Positive selection by categories of evolutionary conservation . . 5
4 A histogram of the number of contigs/isotigs that individual reads are split into by the Newbler assembly. 8
5 Classification of reads by the route they followed into the final combined assembly (MN category only)) 11
6 Contig and read contribution to second-order contigs. 12
7 Span and reference transcriptome coverage (in bases) 14
8 Span and reference transcriptome coverage in percent of proteins hit 15
9 Span and reference transcriptome coverage in percent of proteins coverd to at least 80% of their length 16
10 Agreement of GO-annotation 22

List of Tables

1 Basic statistics for the first-order assemblies and the second-order assembly 9
2 Number of reads, first-order contigs and second-order contigs for different categories of contigs 10
3 example table for assembly-measurements on contigs (as given in A.crassus_transcriptome_contig_data.csv) 19
4 number of contigs with a coverage and unique-coverage of zero, inferred from mapping of raw reads, listed by contig-category . 20

1 Additional figures referenced in the main text

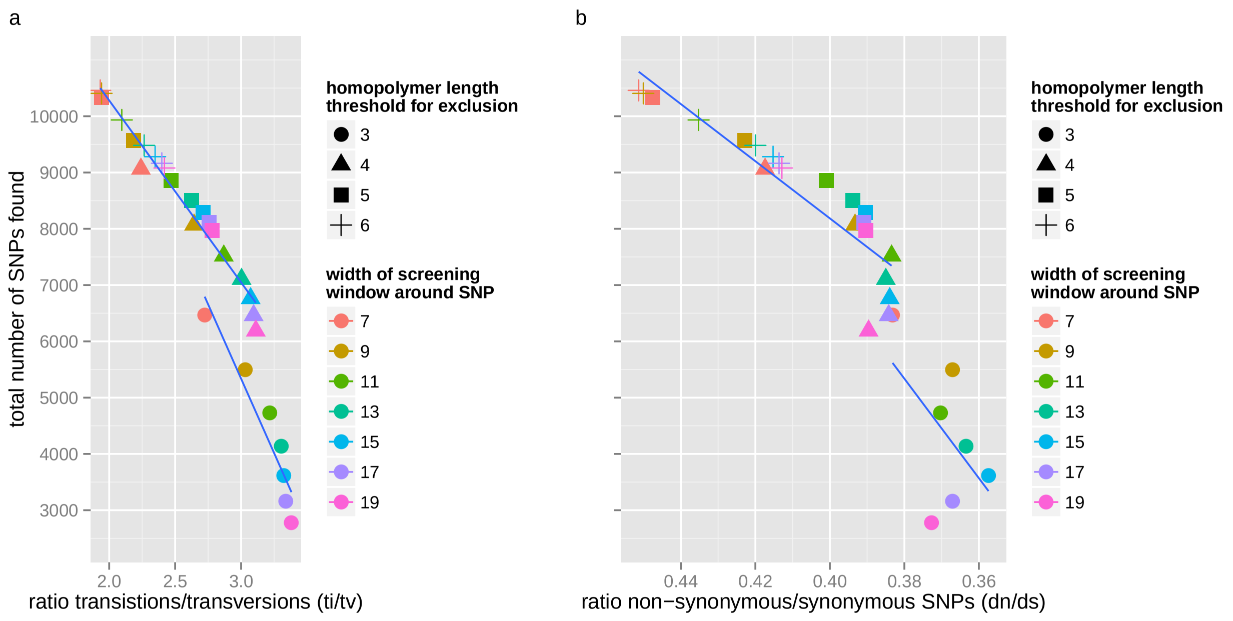

Additional Figure 1: Filtering of putative SNPs. When SNPs in or adjacent to homopolymeric regions are removed changes in ti/tv and dn/ds are observed: as the overall number of SNPs is reduced both ratios change to more plausible values. Note the reversed axis for dn/ds to plot these lower values to the right. For homopolymer length > 3 a linear trend for the total number of SNPs and the two measurements is observed. A width of 11 for the screening window provides most plausible values (suggesting specificity) while still incorporating a high number of SNPs (sensitivity).

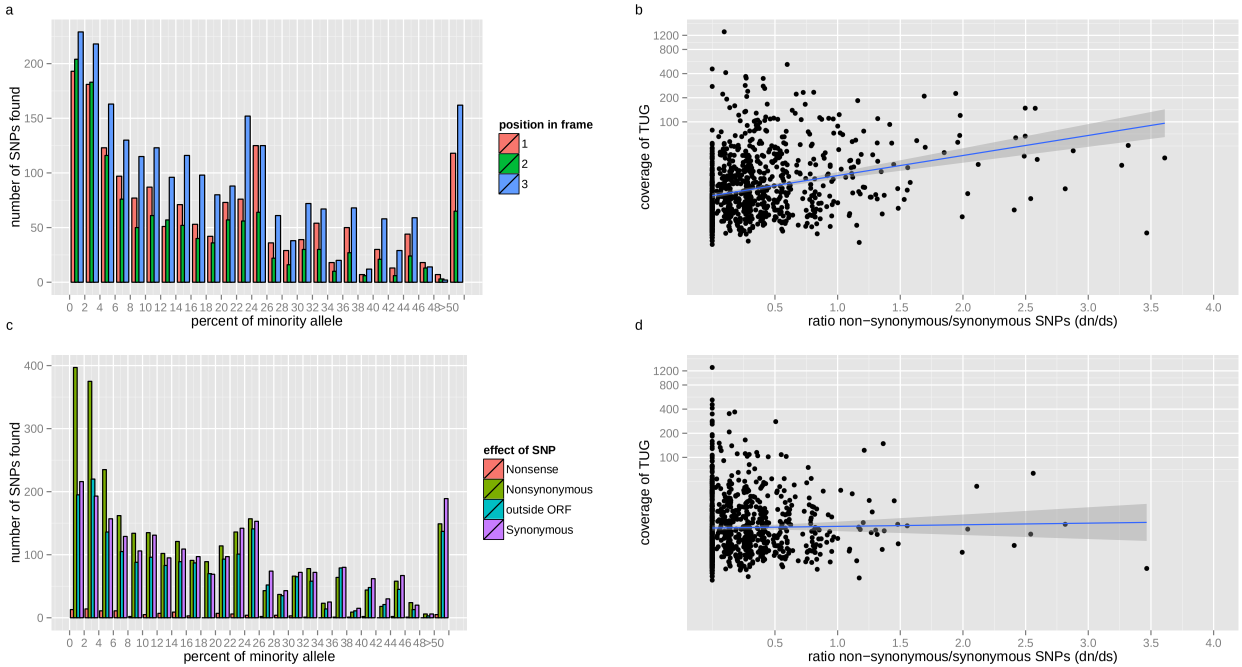

Additional Figure 2: SNP distribution is affected by codon position and coverage. Overabundance of SNPs at codon-position two (a) and of non-synonymous SNPs (c) for low percentages of the minority allele. (b) Significant positive correlation of coverage and dn/ds before removing these SNPs at a threshold of 7% ($p < 0.001$, $R^2 = 0.015$) and (d) absence of such a correlation afterwards (p =0.192, R^2 <0.001).

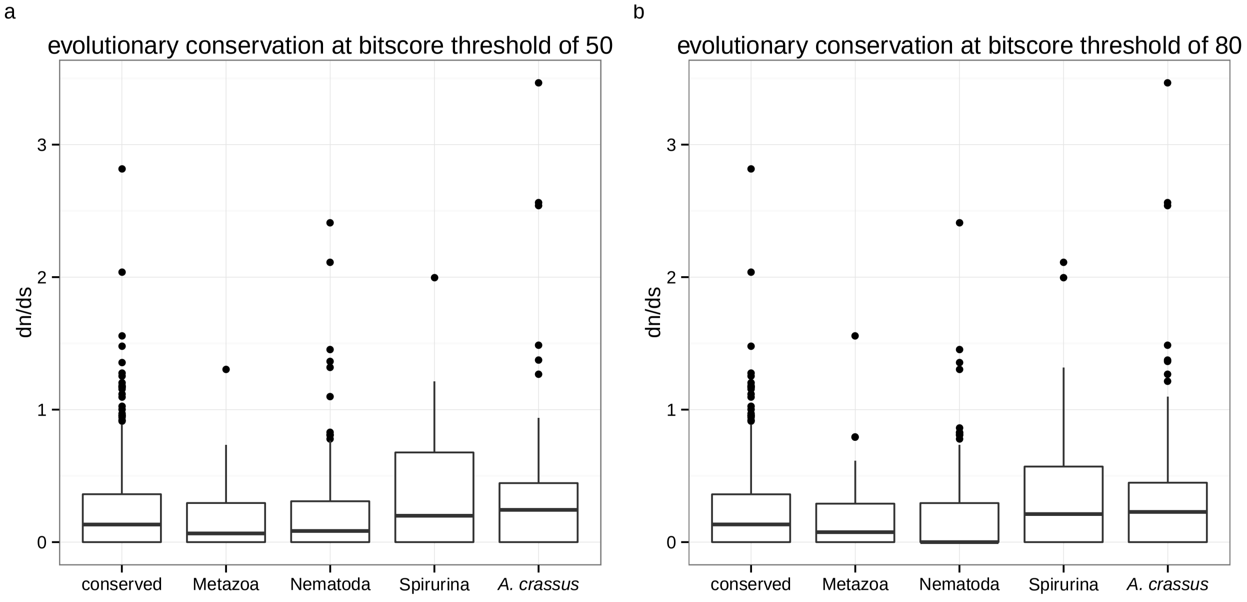

Additional Figure 3: Positive selection by categories of evolutionary conservation. Boxplot of dn/ds based on the screened set of SNPs in TUGs by different categories of evolutionary conservation. Significant comparisons are sequences novel in Metazoa vs. novel in *A. crassus* (0.009 and 0.002; p-value for bitscore of 50 and 80, Nemenyi-Damico-Wolfe-Dunn test), in Nematoda vs. in *A. crassus* (0.03 and 0.009). Sequences novel in Spirurina failed to show significantly elevated dn/ds, despite higher median values due to the low number of contigs of this category with a dn/ds obtained.

2 The transcriptome of *A.crassus*

2.1 Sequencing, trimming and pre-assembly screening

A total of 756,363 raw sequencing reads were generated for *A. crassus* (Table 1). These were trimmed for base call quality, and filtered by length to give 585,949 high-quality reads (spanning 169,863,104 bases). From *An. japonica* liver RNA 159,370 raw reads were generated, and 135,072 retained after basic quality screening. These eel reads were assembled into 10,639 contigs. The *A. crassus* reads were screened for contamination by host sequence by comparison to our assembled *An. japonica* 454 transcriptome and publicly accessible *An. anguilla* sequence data, and 30,071 reads removed. By comparison to *A. crassus* small subunit ribosomal RNA (sequenced previously) and large subunit ribosomal RNA (assembled from our reads in preliminary analyses), 181,783 were tagged and removed. The L2 library proved to have contributions for other cobionts of the eel, and 5,286 reads were removed because they matched closely to cercozoan (likely parasite) ribosomal RNA genes.

3 Evaluating a method of combining assemblies

3.1 Overview

The pre-processed *A. crassus* data-set consisting of 100491819 bases in 353055 reads (58617 generated using "FLX-chemistry", 294438 using "Titanium-chemistry") was assembled following an approach proposed by [1]: two assemblies were generated, one using Newbler v2.6 [2], the other using Mira v3.2.1 [3]. The resulting assemblies (referred to as first-order assemblies) were merged with Cap3 [4] into a combined assembly (referred to as second-order assembly).

3.2 The Newbler first-order assembly

During transcriptome assembly (with options -cdna -urt) Newbler can split individual reads spanning the breakpoints of alternate isoforms, to assemble e.g. the first portion of the reads in one contig and the second portion in two different contigs. Subsequently, multiple so called isotigs are constructed and reported, one for each putative transcript variant. While this approach could be helpful for the detection of alternate isoforms, it also produces short

contigs (especially at error-prone edges of high-coverage transcripts) when the building of isotigs fails. The read status report and the assembly output in ace format the program provides include short contigs only used during the assembly process, but not reported in the contigs file used in transcriptome-assembly projects (454Isotigs.fna). Therefore to get all reads not included in contigs (i.e. a consistent definition of "singleton") it was necessary to add all reads appearing only in contigs not reported in the fasta file to the reported singletons. The number of singletons increased in this step from the 26211 reported to 109052. We later also address the usefulness of Newbler's report vs. the expanded singleton-category, but for the meantime we define singletons as all reads not present in contigs reported from a given assembly.

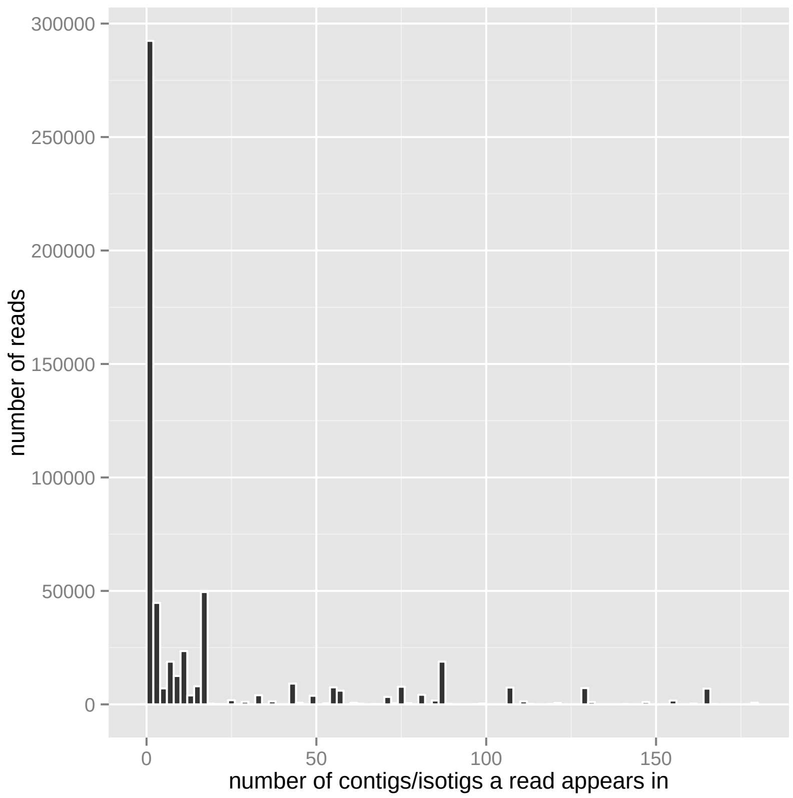

Additional Figure 4: A histogram of the number of contigs/isotigs that individual reads are split into by the Newbler assembly.

While, the splitting of reads in the Newbler assembly can give useful information on possible isoforms, the number of contigs Newbler split one read into (in some cases more than 100 contigs) seems inflated (see additional figure 4). This emphasises the need for further processing of the contigs. The maximum number of read splits in a contig and its usefulness in estimating contig quality will be discussed later in greater detail.

3.3 The Mira assembly and the second-order assembly

The Mira assembly (with options –job=denovo,est,accurate,454) provided a second estimate of the transcriptome. In this assembly individual reads were not split. The number of reads not used in the Mira assembly was 65368.

To combine the two assemblies Cap3 was used with default parameters and the quality information from first-order assemblies was included. Below, we explore how information from both estimates of the transcriptome were

integrated into the final second-order assembly.

	Newbler	Mira	Second-order(MN)
Max length	6300	6352	6377
Number of contigs	15934	22596	14064
Number of Bases	8085922	12010349	8139143
N50	579	579	662
Number of congtigs in N50	4301	6749	3899
non ATGC bases	375	29962	5245
Mean length	508	532	579
Number of singletons	26211/109052	65368	23175/47669
Span of singletons	7022901/31670649	17833358	6108621/13291587

Additional Table 1: Basic statistics for the first-order assemblies and the second-order assembly (for which only the most reliable category of contigs is shown. For Newbler the number of singletons is given as defined above as "unused reads" and as reported. Singletons for the second-order assembly are the intersection of these with the Mira singletons.

Additional table 1 gives summary statistics of the different assemblies. Mira clearly produced the biggest assembly, both in terms of number of contigs and bases). The second-order assembly is slightly smaller than the Newbler assembly. The second-order assembly had on average longer contigs than both first-order assemblies and a higher weighted median contig size (N50).

3.4 Data categories in the second-order assembly

Three main categories of assembled sequence data can be distinguished in the second-order assembly, each one with different reliability and utility in downstream applications:

The first category of data obtained are the singletons of the final second-order assembly. It comprises raw sequencing reads that neither of the first-order assemblers used. It is therefore the intersecion of the Newbler singletons (as defined in 3.2) and the Mira singletons. 47669 reads fell in this category. A second category of sequence is the first-order contigs that were not assembled in the second-order assembly (the singletons in the Cap3 assembly; M_1 and N_1 in additional table 2). Second-order contigs in which first-order contigs from only one assembler are combined (M_n and N_n in additional table 2) were also included in this category. We consider sequences in this category as only moderately reliable, as they are supported by only one assembly algorithm. The category of contigs considered the most reliable contains all second-order contigs with contribution from both first-order assemblies (MN in additional table 2). For this last, most reliable (MN) category, reads contained in the assembly can be categorised depending on whether they entered the assembly via both or only via one first-order assembly.

	M_1	M_n	MN	N_n	N_1
Second order contigs		164	13887	13	
First order contigs	2347	897	mira=19352/Newbler=14410	40	1484
reads	42172	21153	one=269868/both=193308	1538	13100

Additional Table 2: Number of reads, first-order contigs and second-order contigs for different categories of contigs (M_1 and N_1 = first-order contigs not assembled in second-order assembly, from mira and newbler respectively; M_n and N_n = assembled in second-order contigs only with contigs from the same first-order assembly; MN = assembled in second-order contigs with first order contigs from both first order assemblies

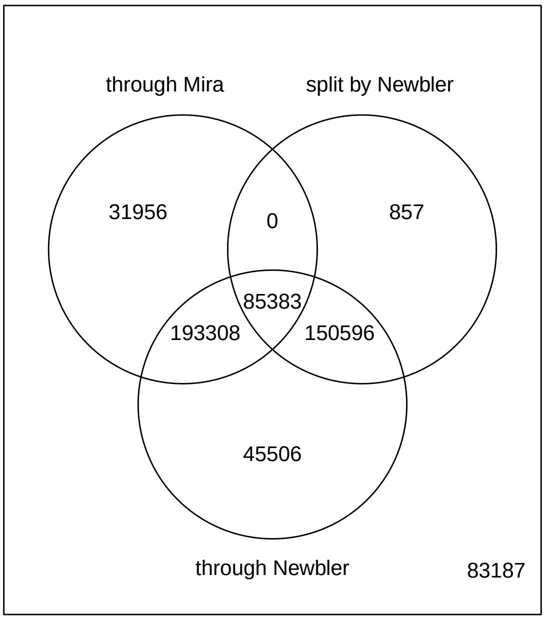

Additional Figure 5: Classification of reads by the route they followed into the final combined assembly (MN category only))

Additional figure 5 gives a more detailed view of the fate of the reads split by Newbler during first-order assembly. Interestingly most reads split by Newbler ended up in the high-quality category of the second order assembly.

3.5 Contribution of first-order assemblies to second-order contigs

Examining the contributions of contigs from each of the assemblies to one second-order contig in additional figure 6a it becomes clear that the Mira assembly had many redundant contigs. The reads contributing to these were assembled into the same contig by Newbler and finally also into one second-order contig by Cap3.

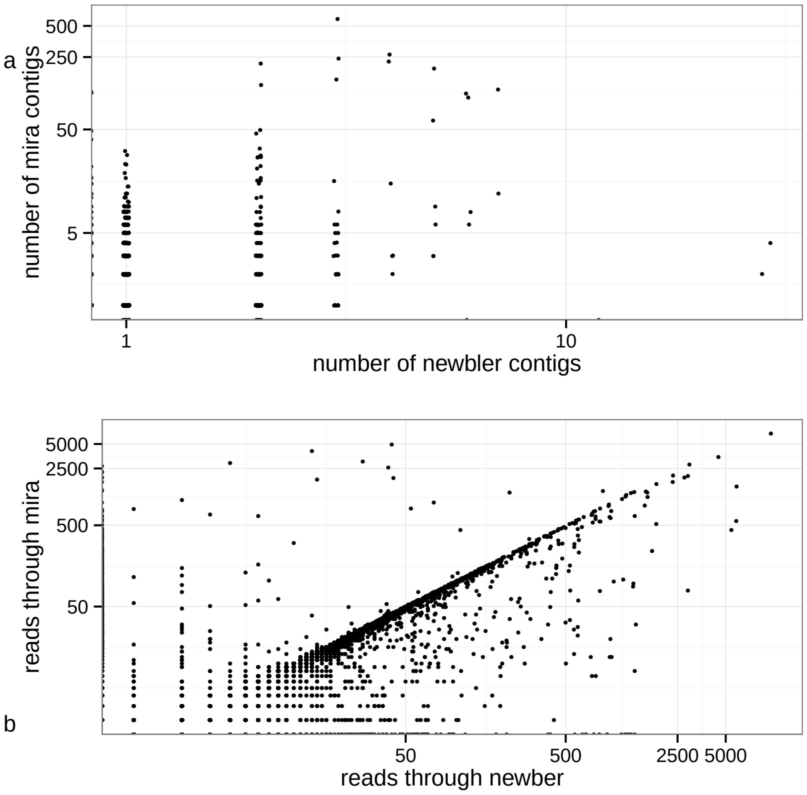

Additional Figure 6: Contig and read contribution to second-order contigs. (a) Number of first-order contigs from both first-order assemblies for each second order contig. (b) Number of reads through Newbler and Mira for each second-order contig.

A different picture emerges from examination of the contribution of reads through each of the first-order assemblies (additional figure 6b). Here, for most second-order contigs, many more reads are contributed through Newbler contigs. This is because Newbler has more reads summed over all contigs, due to the duplication resulting from splitting of reads.

3.6 Evaluation of the assemblies

To further compare assemblies (Mira or Newbler first-order assemblies including or excluding their singletons) and the second-order assembly (including different contig categories and singletons) we evaluated the number of bases or proteins their contigs and singletons (partially) cover in the related model nematodes, *Caenorhabditis elegans* and *Brugia malayi.* For this purpose we used BLAST (blastx E-value cut-off 1e-5) and a custom perl script provided by S. Kumar (mask_blast.pl available from github.com/sujaikumar/assemblage).

In addition, the span of an assembly can give an indication of redundancy or artificially assembled data. If span increases without reference coverage, the dataset is likely to contain more redundant or artificial information, and a more parsimonious assembly should be preferred. The coverage for the two reference species was plotted against the size of the assembly dataset to estimate the completeness conditional on the size of the assembly (additional figures 7, 8, 9).

From the assemblies excluding singletons (in the lower left corners of additional figures 7, 8 and 9; with lower size and database coverage) the highly reliable second order assembly contigs produced the highest per-base coverage in both reference species, with the Newbler assembly second and Mira producing the lowest reference coverage. When the contigs considered lower quality (i.e. supported by only one assembler) were added to the second-order assembly, reference coverage increased moderately.

A comparison of the addition of only Newbler's reported singletons and the addition of all singletons to the Newbler assembly showed that the reported singletons increased reference coverage to the same extent as all singletons, while the non-reported singletons only increased the size of the assembly. It can be concluded that the latter contain very little additional biological information and are likely to include only error-prone or variant reads.

The second-order assembly including the intersection of first-order singletons had similar reference coverage metrics to the Newbler assembly, but was larger in size. Adding the less reliable set of one-assembler supported second-order contigs the assembly covered the most bases in both references. When the intersection of the Newbler "reported singletons" and Mira singletons was added a parsimonious assembly with high reference coverage (termed fullest assembly; and labeled FU in additional figures 7, 8 and 9) was obtained.

When database proteins covered for at least 80% of their length are considered (additonal figure 9) the second-order assembly was superior: Both ex- and in-cluding singletons the second-order assembly outperformed the first-order assemblies. Moderate gains in reference coverage were made again

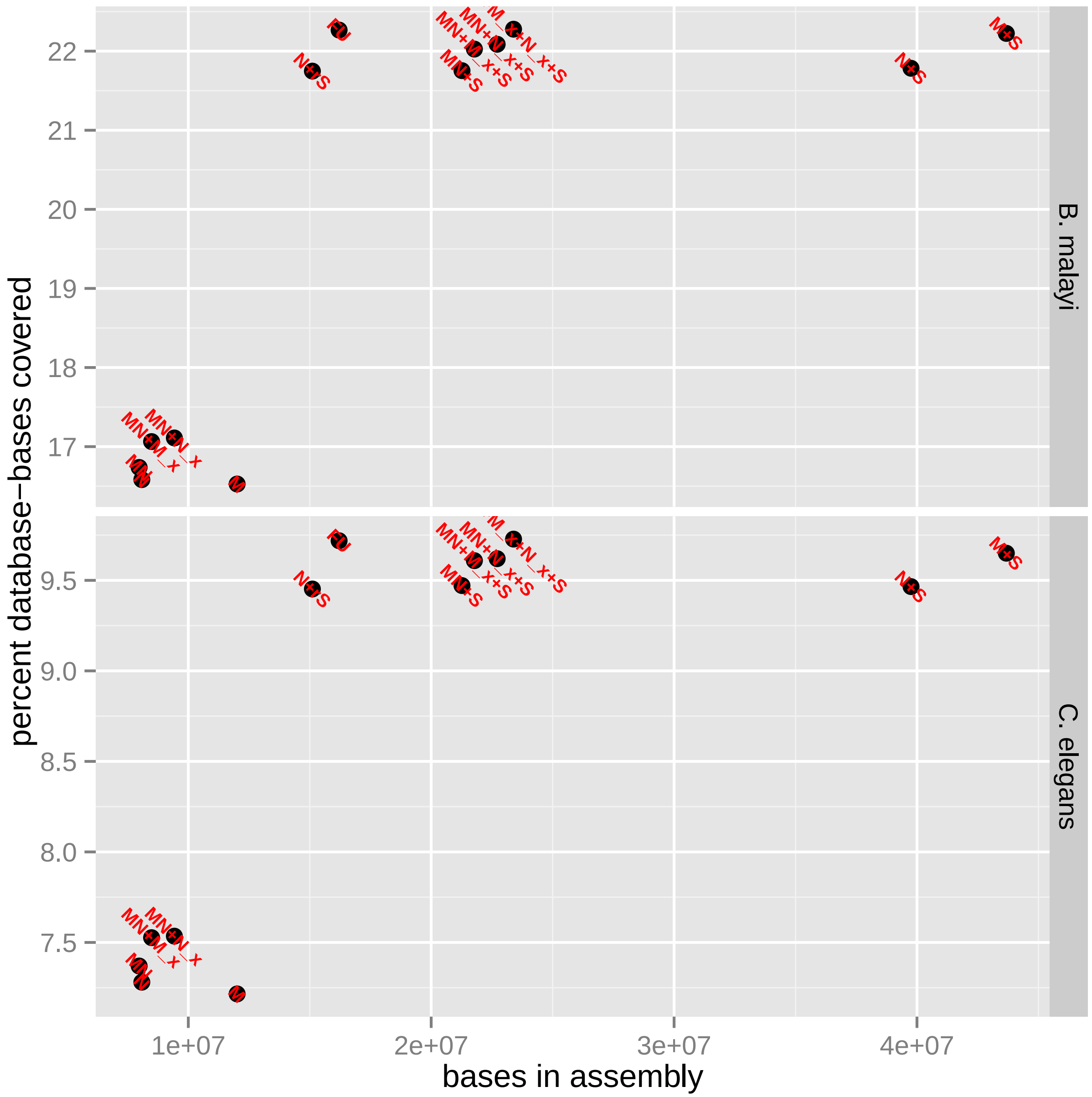

Additional Figure 7: Span and reference transcriptome coverage (in bases) for different assemblies and assembly-combinations. M = Mira; N = Newbler; $M + S$ = Mira + singletons; $N + S$ = Newbler plus singletons; $N + Sr$ = Newbler plus singletons reported in readstatus.txt; MN = second-order contigs supported by both first-order; $MN + N\_x$ = second-order MN plus contigs only supported by Newbler ($N\_x = N\_n$ and N_1); $MN + M\_x$ = same for Mira-first-order-contigs; $MN + M\_x + S$ and $MN + N\_x + S$ same with singletons; FU = second-order contigs supported by both or one assembler plus the intersection of Newbler reported singletons and Mira-singletons = the basis for the “fullest assembly” used in later analyses.

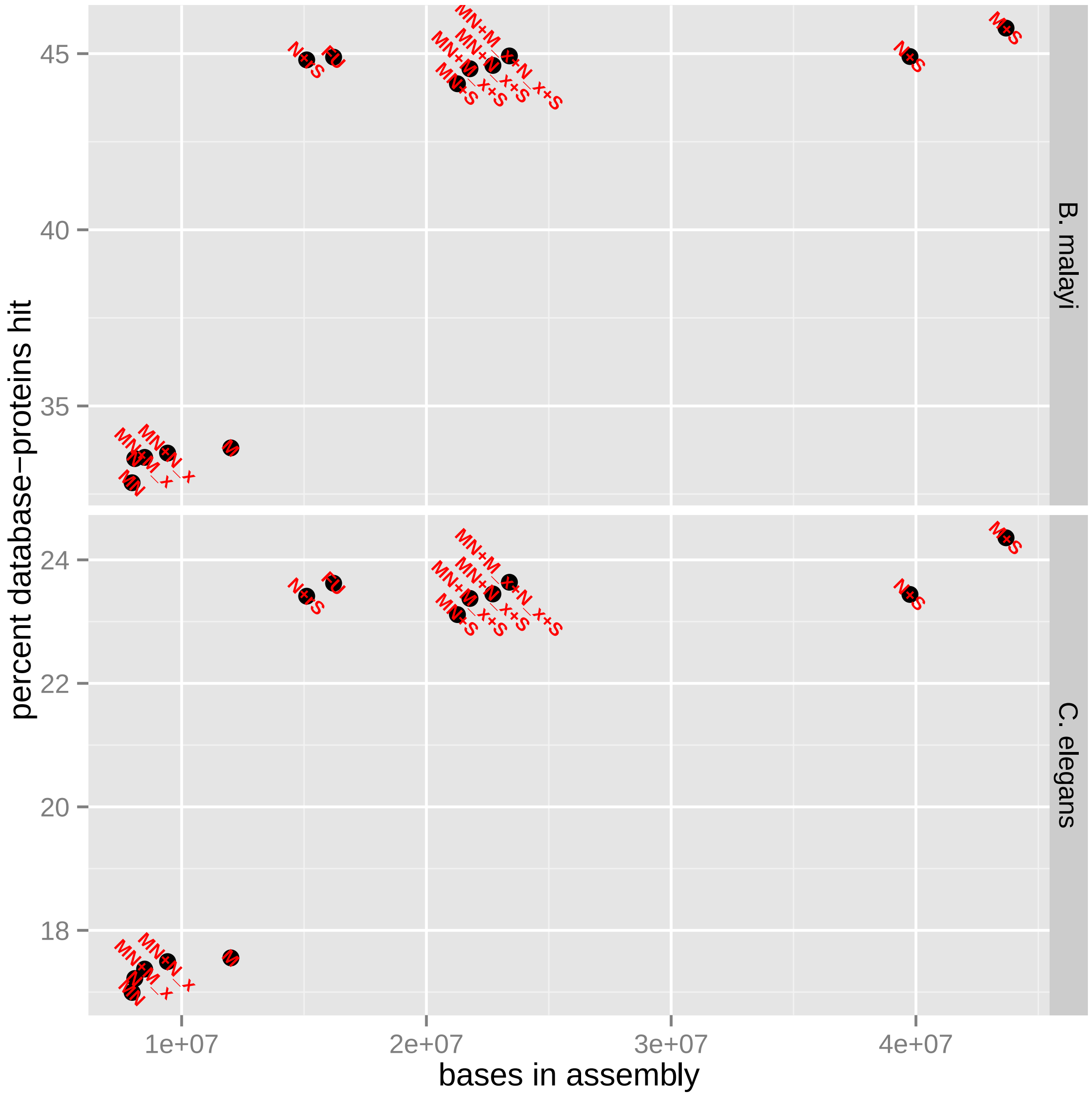

Additional Figure 8: Span and reference transcriptome coverage in percent of proteins hit for different assemblies and assembly-combinations. For category abrevations see additional figure 7.

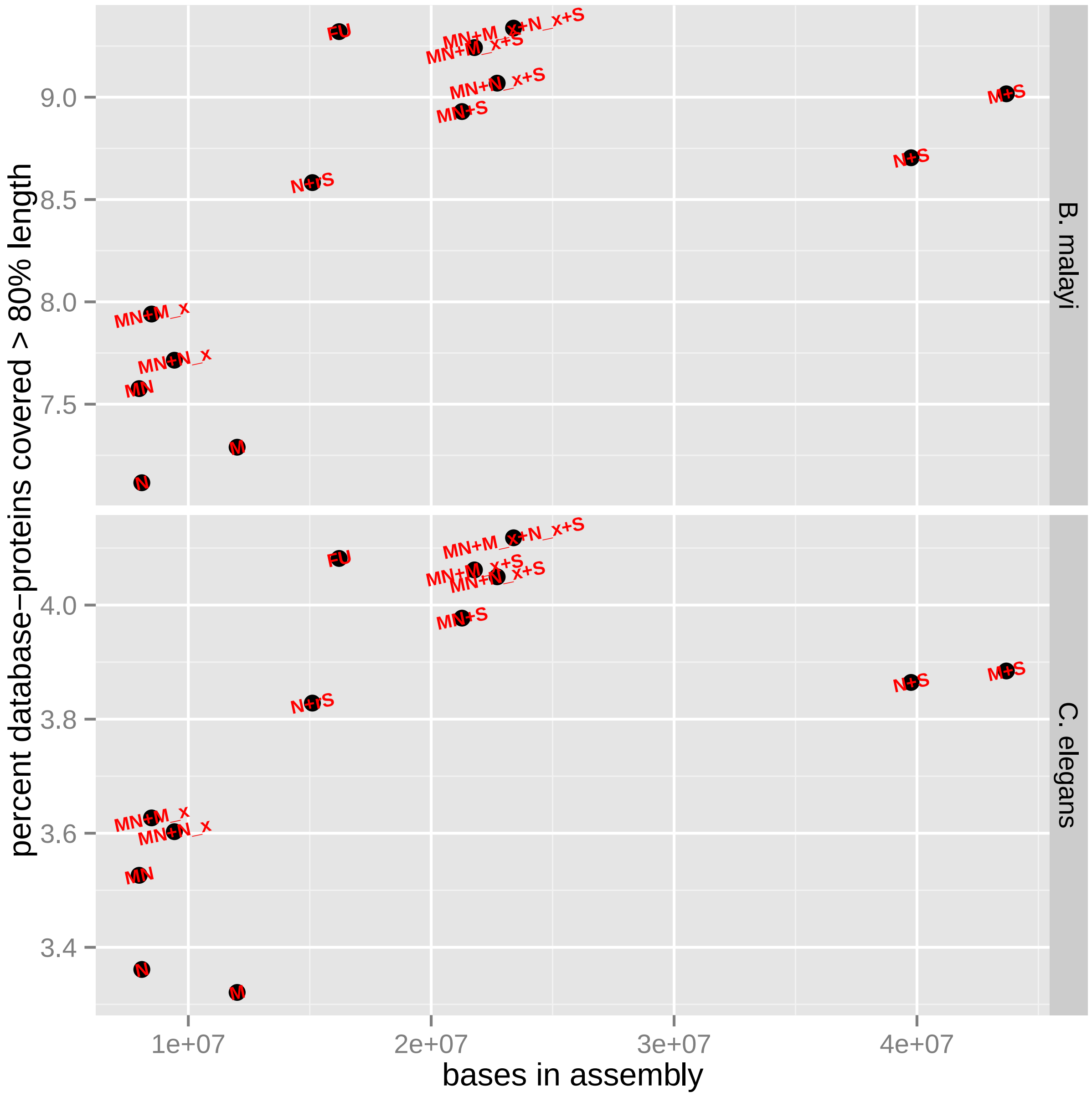

Additional Figure 9: Span and reference transcriptome coverage in percent of proteins coverd to at least 80% of their length for different assemblies and assembly combinations. For category abrevations see additional figure 7.

for the addition of dubious single assembler supported second-order contigs.

Given this evaluation we defined the "minimal adequate assembly" as the subset of contigs of the second-order assembly supported by both assemblers (labeled MN above). Given the performance of the singletons Newbler reported, we defined the "fullest assembly" as all second-order contigs (including those supported by only one assembler) plus the intersection of reported Newbler singletons and Mira singletons.

The proportion of proteins covered in *C. elegans* by *B. malayi* coding sequences was with 26.85% simimlar to our estimate for the fullest (FU) assembly. The proportion of bases (25.46%) and of proteins coverd to at least 80% of their length (14.67%) was however higher for the coding sequences from *B. malayi*.

3.7 Measurments on second-order assembly

Based on the tracking of reads through the assembly process, we calculated the following statistics for each contig in the second-order assembly.

- number of Mira and Newbler first-order contigs
- number of reads in each contributing Mira contig and Newbler contig
- number of reads split by Newbler in first-order assembly
- number of read-split events in the first-order assembly (the sum of reads multiplied by number of contigs a read has been split into)
- maximal number of first-order contigs a read in the contig has been split into during Newbler assembly
- the number of reads same-read-paires from the Newbler and Mira first order-assembly merged in a second order contig
- cluster-id of the contig: the cluster to which the contig belongs (contigs "connected" by sharing reads are placed in one cluster; similar to the graph clustering reported in [5]).
- number of other second-order contigs containing the same read i.e. the size of the cluster

3.7.1 Contig coverage

As well defined coverage-information was not readily avaiable from the output of this combined assembly aproach (although we followed individual reads through the process) we inferred coverage by mapping the reads used for assembly against the fullest assembly using ssaha2 [6] with parameters (-kmer 13 -skip 3 -seeds 6 -score 100 -cmatch 10 -ckmer 6 -output sam -best 1). This read mapping was summarised by:

- mean per base coverage
- mean unique per base coverage

The ratio of mean per base coverage and unique per base coverage (the standard for assessing coverage) can be used to assess the redundancy of a contig.

3.7.2 Example use of the contig-measurements

Based on these measurements the generation of a given contig by the assembly process can be reconstructed. Additional table 3 gives an excerpt of the contig-measurements reported in additional file A.crassus_transcriptome_contig_data.csv. The example contigs are all from large contig clusters (cluster.size), where interpretation of the assembly history is more complex:

Contig1047 is from the well trusted MN category of contigs. It consists of only one contig from each first-order assembly (newbler_contigs and mira_contigs), each containing a set of reads of moderate size: 16 from Newbler (reads_through_newbler), and 26 from Mira (reads_through_mira). 8 of the 16 reads Newbler used were also assembled to a differnt Newbler contig (num.new.split). That each of the 8 reads only appeared in one other Newbler contig is evident as the number of split events is 16 (sum.new.split) and the maximal number of splits for one read is 2 (max.new.split). 13 (num.SndO.pair) same-read-pairs from the two different first-order assemblies were merged in this second-order contig, leaving 3 (16-13) reads in Newbler contigs and 13 (26-13) reads in Mira contigs, which all could potentially have ended up in other contigs. The contig is in a cluster (CL62), which contains 24 contigs (cluster.size). The complete graph structure linking these 24 contigs cannot be reconstructed from this contig summary data. The summary data makes clear the sources of links for cluster-affiliation: In the case of Contig1047 from 3 and 13 unlinked read-paires from both first-order assemblies and 8 split reads from Newbler first-order contigs.

A comprehensive interpretation of the other example contigs depicted is left to the reader. It should just be remarked, that in case of one-assembler

	Contig1047	Contig10719	Contig104	Contig13672
reads_through_Newbler	16	1351	0	14
reads_through_Mira	26	651	135	0
Newbler_contigs	1	5	0	2
Mira_contigs	1	9	4	0
category	MN	MN	M_n	N_n
num.new.split	8	1314	0	0
sum.new.split	16	2628	0	0
max.new.split	2	2	0	0
num.SndO.pair	13	644	0	0
cluster.id	CL62	CL6	CL176	CL235
cluster.size	24	18	5	5
coverage	4.200342	267.495458	41.003369	2.920755
uniq_coverage	4.248960	7.425507	2.568000	1.196078

Additional Table 3: example table for assembly-measurements on contigs (as given in A.crassus_transcriptome_contig_data.csv)

supported contigs, all reads in that contig could potentially be represented in other contigs, making average cluster-size in these contigs bigger than in MN category.

Interesting measurements calculated for each contig include the cluster membership and cluster size. Such clusters represent close paralogs, duplicated genes, isoforms from alternative splicing or allelic variants. These measurements can be used in later analysis to e.g. reevaluate the likelihood of misassembly in a given set of biologically relevant contigs. All gene sets discussed in the main text were, as a matter of routine, controlled for unusual patterns in the contig meta-data.

3.8 Finalising the fullest assembly set

In order to minimize the amount of sequence with artificially inferred isoform breakpoints we used the unique mapping information described above to detect contigs and singletons not supported by any independent raw data (reads). Additional table 4 gives a summary of these unsupported data by contig category. For all downstream analyses we removed all the MN-category contigs and the contigs (and singletons) from other categories having no unique coverage.

	singletons	M_1	M_n	MN	N_1	N_n
coverage == 0	546	34	2	36	158	0
unique coverage == 0	584	48	2	42	210	3

Additional Table 4: number of contigs with a coverage and unique-coverage of zero, inferred from mapping of raw reads, listed by contig-category

The remaining 40,187 tentative unique genes (TUGs), define the "fullest assembly". The MN-category of contigs form the high credibility assembly (highCA) and the M_n, N_n, M_1, N_1 and Newbler's reported singletons are the additional low credibility assembly (lowCA).

3.9 Scrutinising the GO annotation

GO-annotations based on similarity searches are often considered unreliable for species diverged from the model-species in which they were developed. To investigate the validity of GO terms assigned by BLAST similarity we compared the annot8r derived terms with domain-based (Interpro-Scan) annotations. We classified contigs and their annotations as "in complete agreement" if the lowest level term (the highest number of terms away from the roots "biological process or molecular function") was the same for the two methods. A contig was classified as having a "more detailed" annotation if the respective method yielded a term agreeing at a lower (offspring) level compared to the other. Finally there were contigs for which only one method yielded an annotation.

This comparison revealed high agreement between annotation based on sequence similarity and on domain architecture, where annotations from both sources were present. Half of our contigs, however, were annotated based on similarity alone. It cannot be excluded that these annotations have additional bias not observed in contigs with annotations from both sources. Therefore we did not rely on single detailed GO assignments, but rather searched for overall patterns through enrichment analyses. These analyses make it more feasible to detect a signal at higher levels of annotation, thus alleviating the quality problems of more detailed annotations.

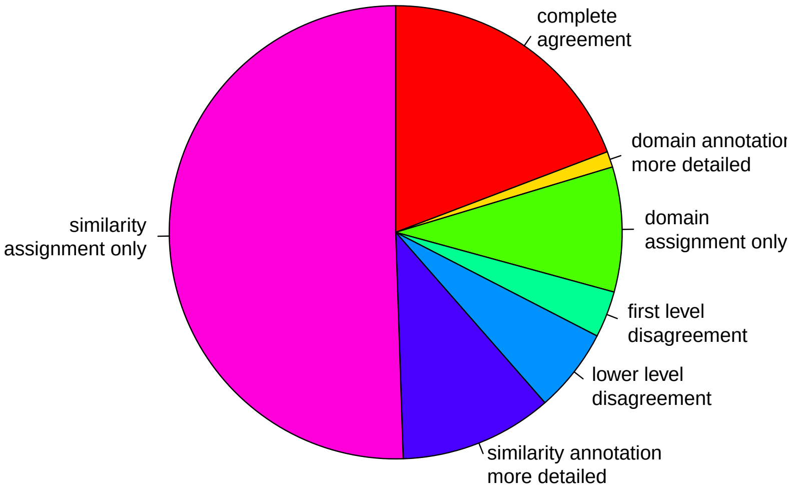

Additional Figure 10: Comparing BLAST with domain based annotations, contigs were classified being annotated in ”complete agreement” if one lowest level term was the same for the two methods. If a lower level term was derived for any of the methods the “more detailed” classification was used. Contigs with disagreeing annotations were grouped into “lower level disagreement” or “first level disagreement” according to annotations at the first (most general) level below the root term.

References

[1] Kumar S, Blaxter ML: **Comparing de novo assemblers for 454 transcriptome data**. *BMC Genomics* 2010, **11**:571, [http://dx.doi.org/10.1186/1471-2164-11-571].

[2] Margulies M, Egholm M, Altman WE, Attiya S, Bader JS, Bemben LA, Berka J, Braverman MS, Chen YJ, Chen Z, Dewell SB, Du L, Fierro JM, Gomes XV, Godwin BC, He W, Helgesen S, Ho CH, Ho CH, Irzyk GP, Jando SC, Alenquer ML, Jarvie TP, Jirage KB, Kim JB, Knight JR, Lanza JR, Leamon JH, Lefkowitz SM, Lei M, Li J, Lohman KL, Lu H, Makhijani VB, McDade KE, McKenna MP, Myers EW, Nickerson E, Nobile JR, Plant R, Puc BP, Ronan MT, Roth GT, Sarkis GJ, Simons JF, Simpson JW, Srinivasan M, Tartaro KR, Tomasz A, Vogt KA, Volkmer GA, Wang SH, Wang Y, Weiner MP, Yu P, Begley RF, Rothberg JM: **Genome sequencing in microfabricated high-density picolitre reactors**. *Nature* 2005, **437**:376–380, [http://dx.doi.org/10.1038/nature03959].

[3] Chevreux B, Pfisterer T, Drescher B, Driesel AJ, Muller WE, Wetter T, Suhai S: **Using the miraEST assembler for reliable and automated mRNA transcript assembly and SNP detection in sequenced ESTs**. *Genome Res.* 2004, **14**:1147–1159, [http://www.ncbi.nlm.nih.gov/pmc/articles/PMC419793].

[4] Huang X, Madan A: **CAP3: A DNA sequence assembly program**. *Genome Res.* 1999, **9**:868–877, [http://www.ncbi.nlm.nih.gov/pmc/articles/PMC310812].

[5] Schwartz TS, Tae H, Yang Y, Mockaitis K, Van Hemert JL, Proulx SR, Choi JH, Bronikowski AM: **A garter snake transcriptome: pyrosequencing, de novo assembly, and sex-specific differences**. *BMC Genomics* 2010, **11**:694, [http://www.ncbi.nlm.nih.gov/pubmed/21138572].

[6] Ning Z, Cox AJ, Mullikin JC: **SSAHA: a fast search method for large DNA databases**. *Genome Res.* 2001, **11**:1725–1729, [http://www.ncbi.nlm.nih.gov/pmc/articles/PMC311141].
