# Supplementary material for: The transcriptome of the invasive eel swimbladder nematode parasite Anguillicola crassus
Source: BMC Genomics. 2013 Feb 8;14:87. doi: 10.1186/1471-2164-14-87 (PMC3630068; doi:10.1186/1471-2164-14-87)

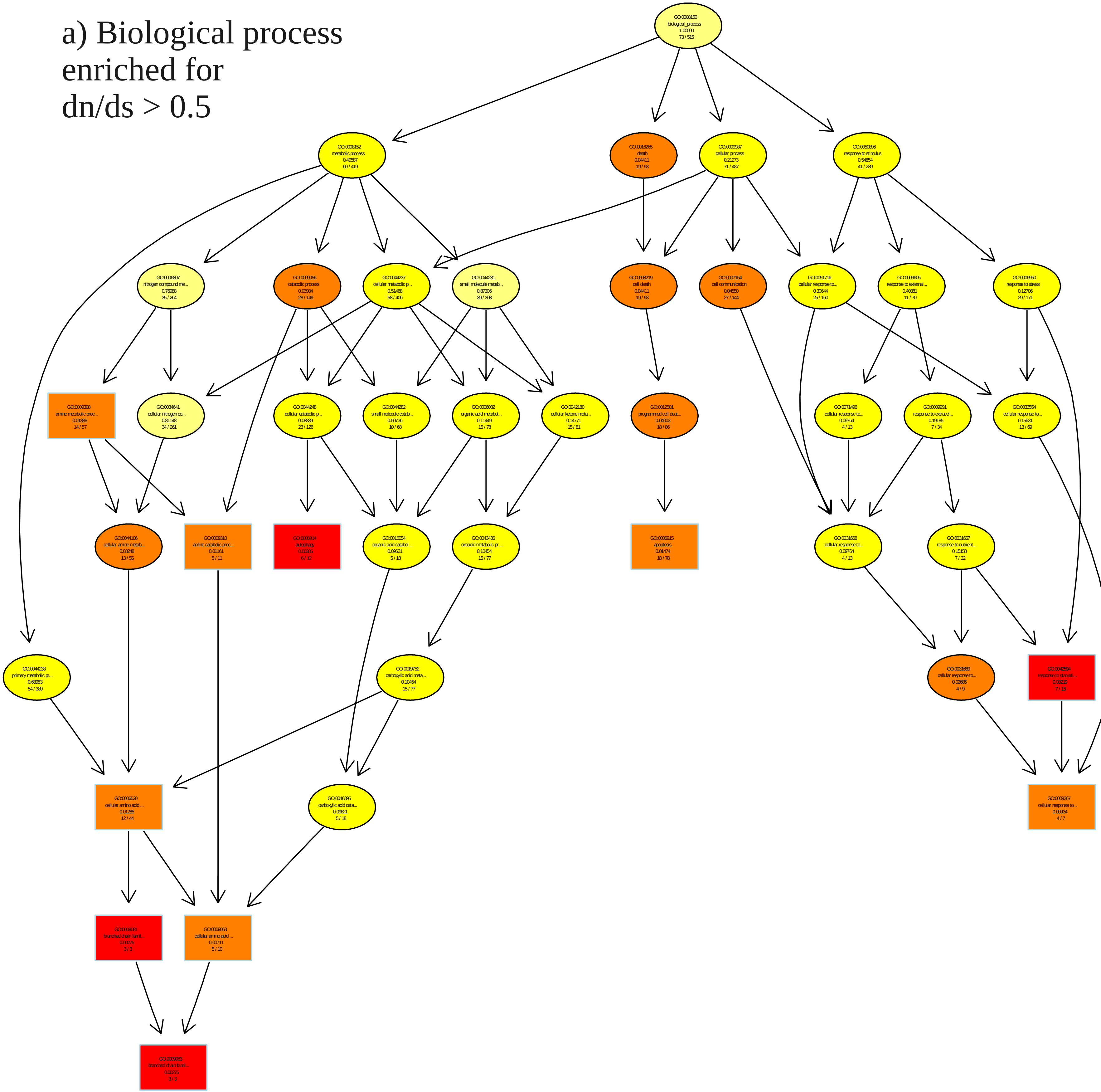
a) Biological process enriched for dn/ds > 0.5
GO:0008150 biological_process 1.00000 73 / 515
GO:0008152 metabolic process 0.49587 60 / 419
GO:0016265 death 0.04411 19 / 93
GO:0009987 cellular process 0.21273 71 / 487
GO:0050896 response to stimulus 0.54854 41 / 289
GO:0006807 nitrogen compound me... 0.76988 35 / 264
GO:0009056 catabolic process 0.03984 28 / 149
GO:0044237 cellular metabolic p... 0.51468 58 / 406
GO:0044281 small molecule metab... 0.87306 39 / 303
GO:0008219 cell death 0.04411 19 / 93
GO:0007154 cell communication 0.04550 27 / 144
GO:0051716 cellular response to... 0.30644 25 / 160
GO:0009605 response to external... 0.40381 11 / 70
GO:0006950 response to stress 0.12706 29 / 171
GO:0009308 amine metabolic proc... 0.01888 14 / 57
GO:0034641 cellular nitrogen co... 0.81148 34 / 261
GO:0044248 cellular catabolic p... 0.08839 23 / 126
GO:0044282 small molecule catab... 0.50736 10 / 68
GO:0006082 organic acid metabol... 0.11449 15 / 78
GO:0042180 cellular ketone meta... 0.14771 15 / 81
GO:0012501 programmed cell deat... 0.04003 18 / 86
GO:0071496 cellular response to... 0.09764 4 / 13
GO:0009991 response to extracel... 0.19185 7 / 34
GO:0033554 cellular response to... 0.15631 13 / 69
GO:0044106 cellular amine metab... 0.03248 13 / 55
GO:0009310 amine catabolic proc... 0.01161 5 / 11
GO:0006914 autophagy 0.00305 6 / 12
GO:0016054 organic acid catabol... 0.09621 5 / 18
GO:0043436 oxoacid metabolic pr... 0.10454 15 / 77
GO:0006915 apoptosis 0.01474 18 / 78
GO:0031668 cellular response to... 0.09764 4 / 13
GO:0031667 response to nutrient... 0.15158 7 / 32
GO:0044238 primary metabolic pr... 0.68983 54 / 389
GO:0019752 carboxylic acid meta... 0.10454 15 / 77
GO:0031669 cellular response to... 0.02685 4 / 9
GO:0042594 response to starvati... 0.00219 7 / 15
GO:0006520 cellular amino acid ... 0.01285 12 / 44
GO:0046395 carboxylic acid cata... 0.09621 5 / 18
GO:0009267 cellular response to... 0.00934 4 / 7
GO:0009081 branched chain famil... 0.00275 3 / 3
GO:0009063 cellular amino acid ... 0.00711 5 / 10
GO:0009083 branched chain famil... 0.00275 3 / 3

b) Cellular compartment enriched for dn/ds > 0.5

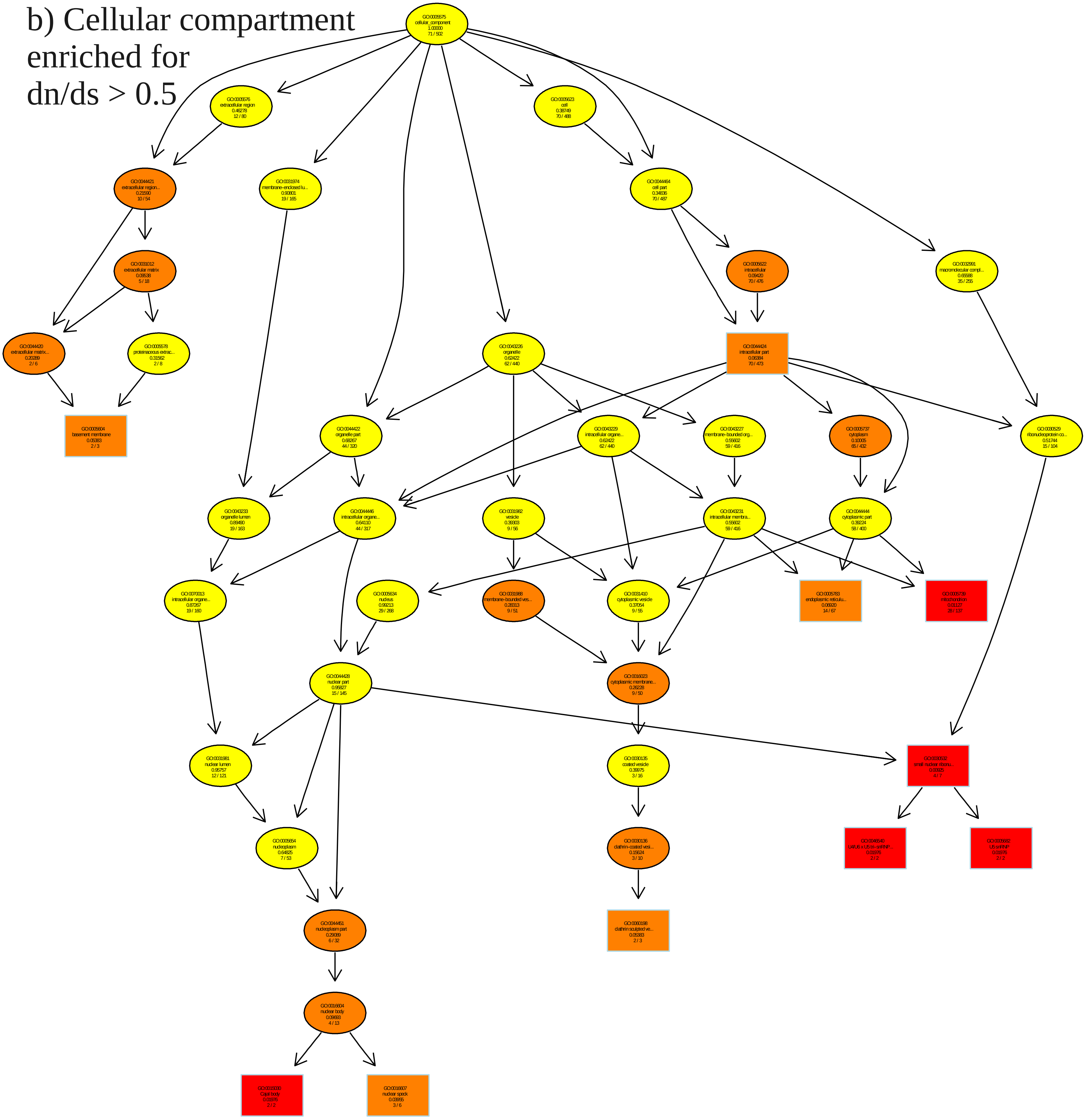

c) Molecular function enriched for dn/ds > 0.5

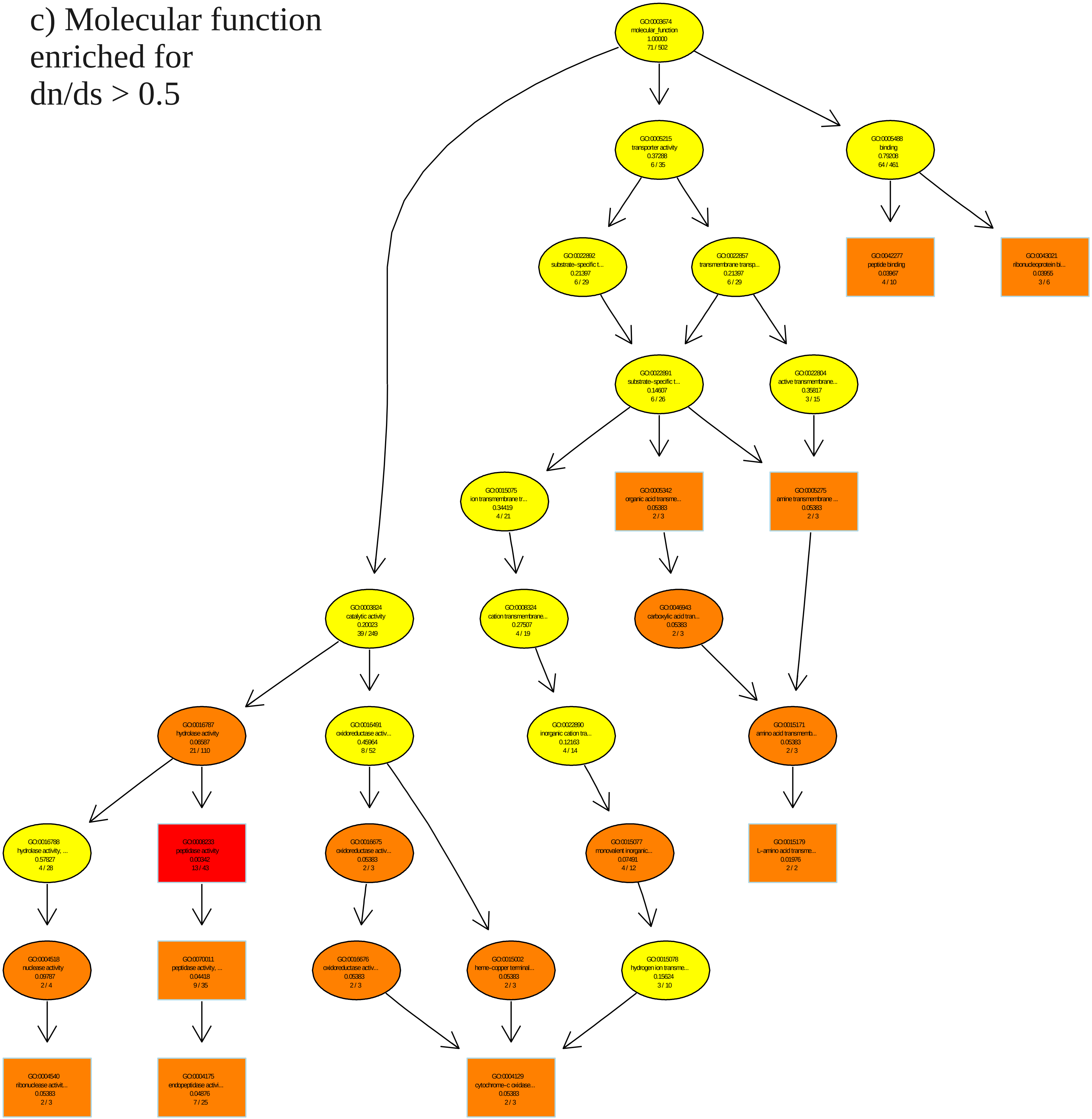

d) Biological process enriched for contigs differently expressed between Asia/Europe

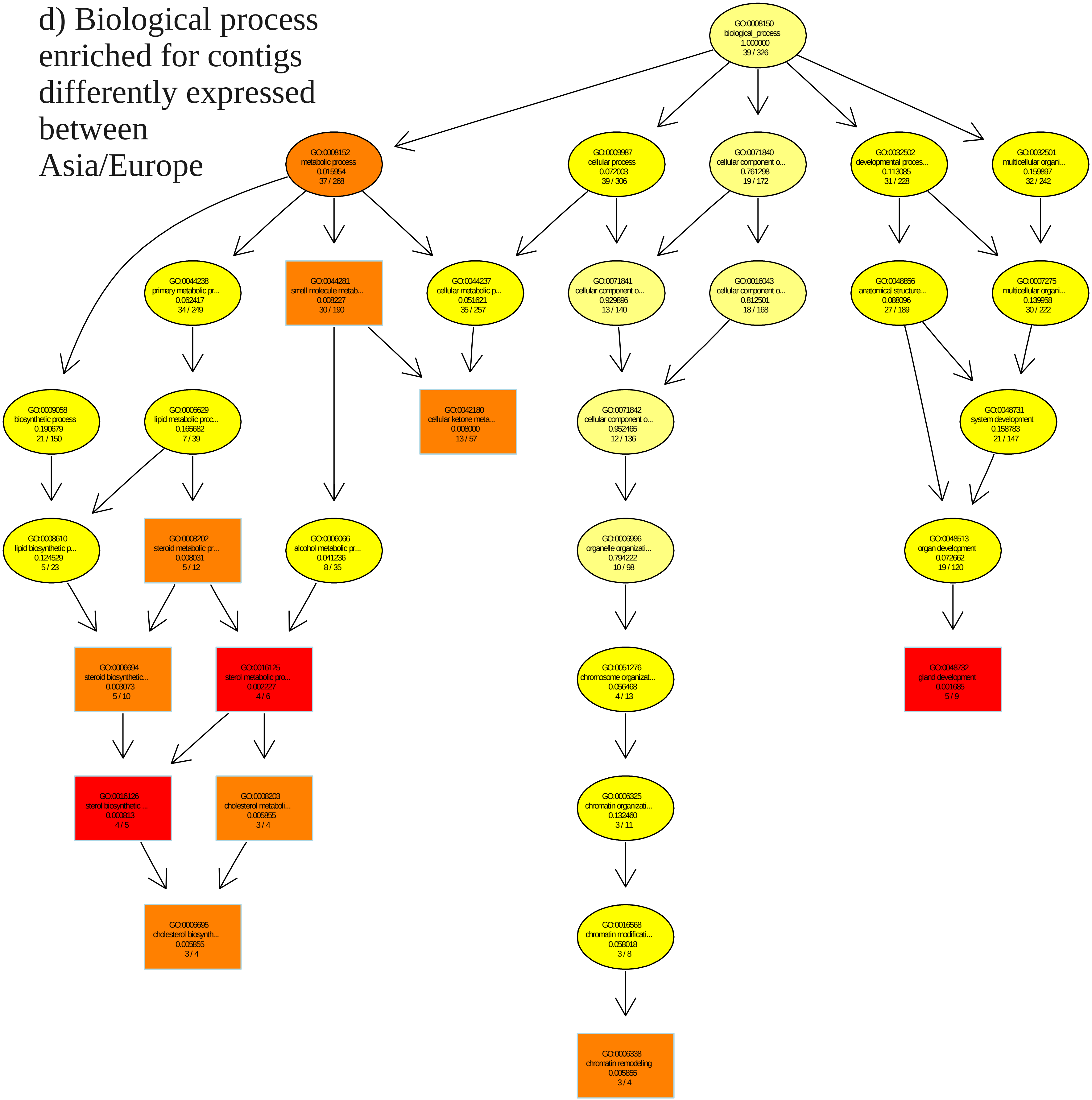

e) Cellular compartment enriched for contigs differently expressed between Asia/Europe

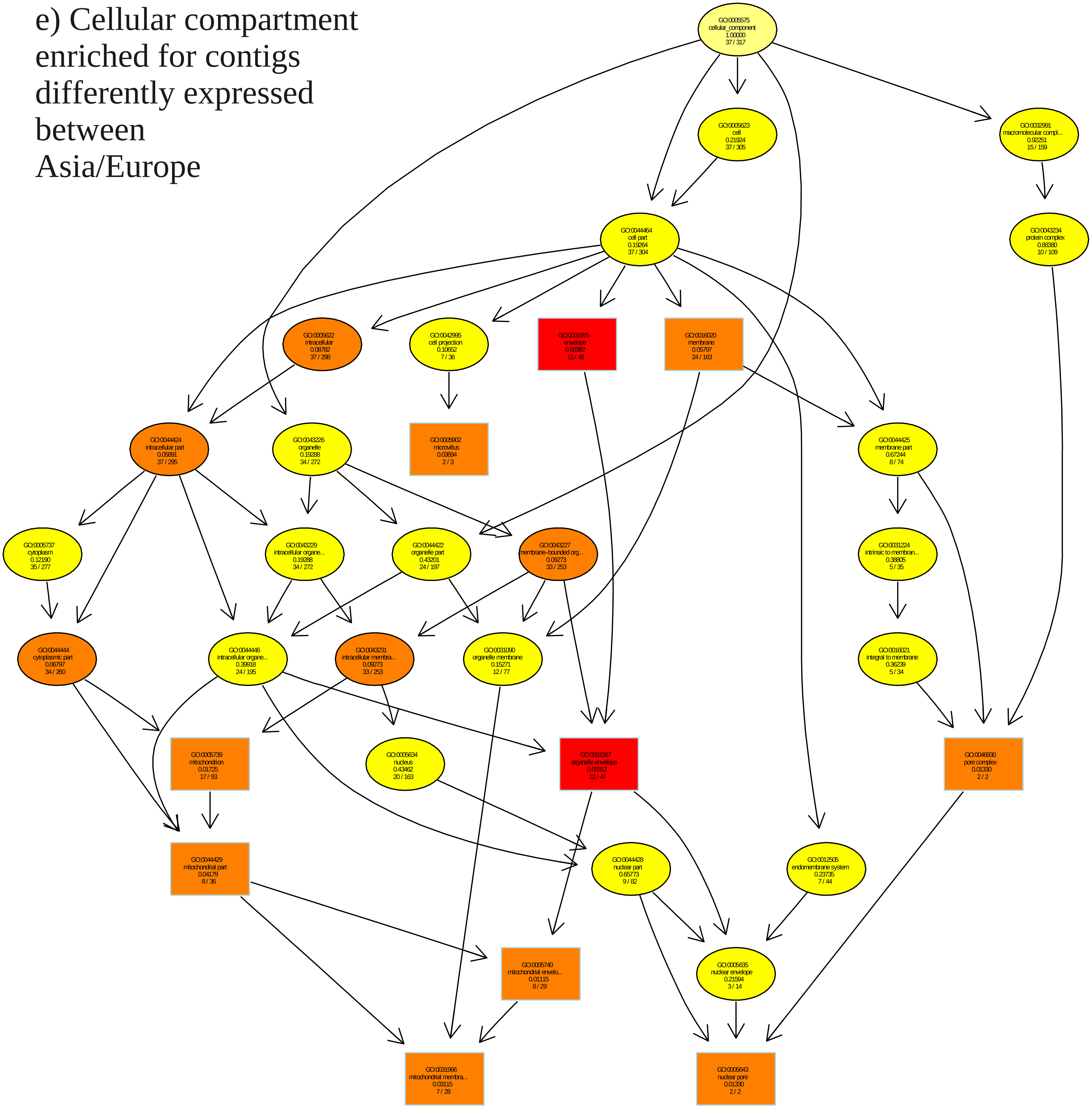

f) Molecular function enriched for contigs differently expressed between Asia/Europe

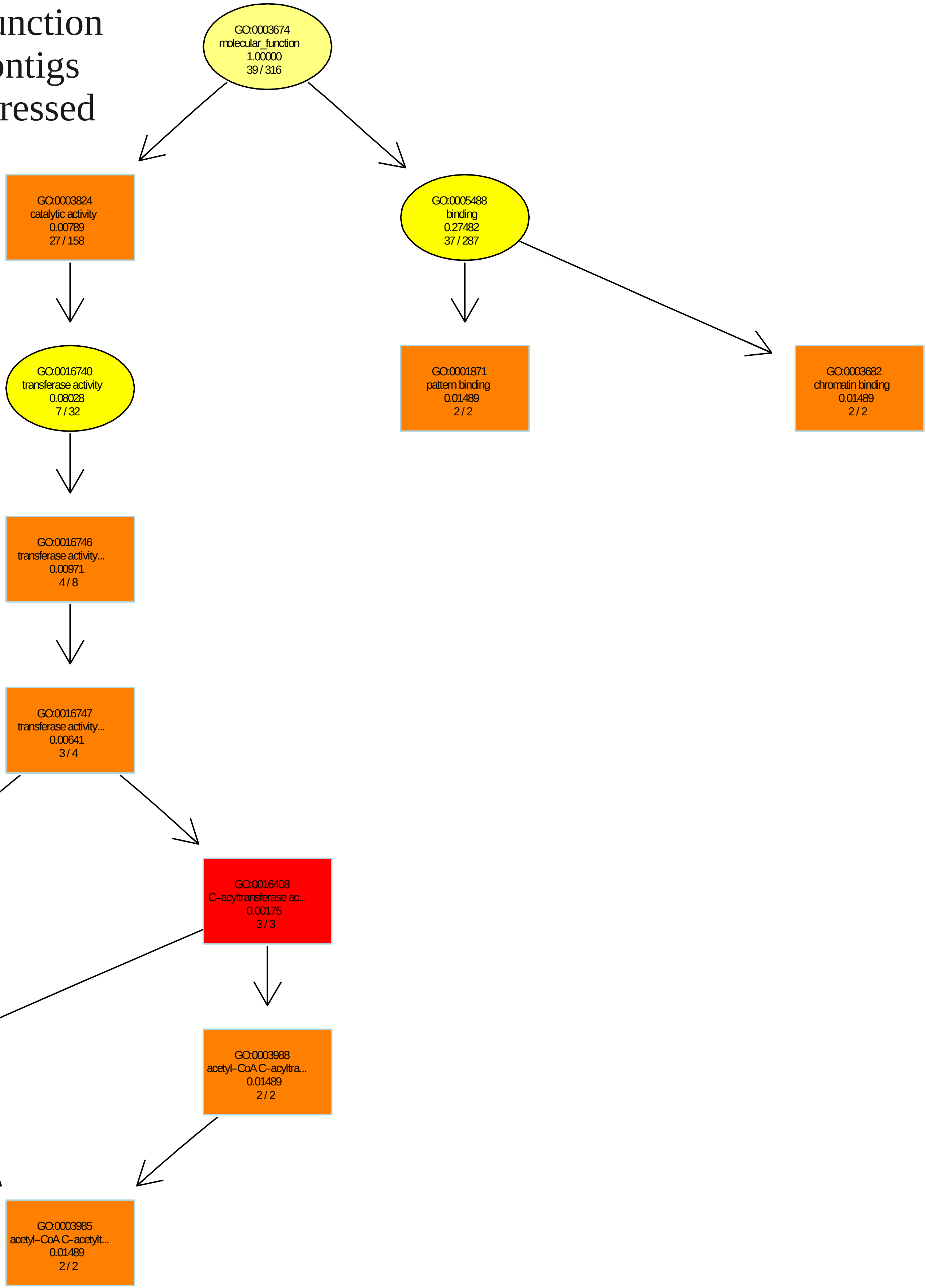

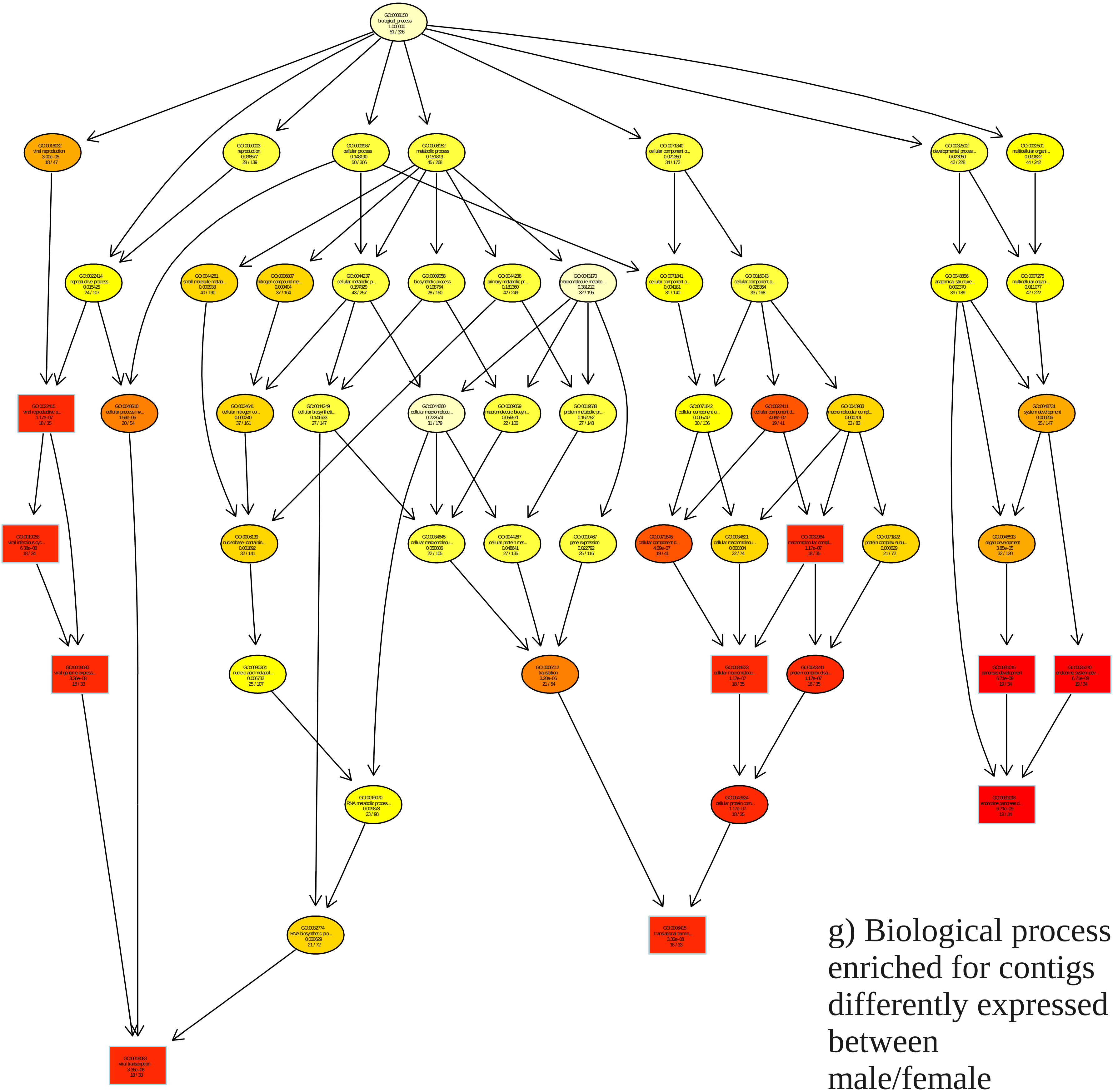

g) Biological process enriched for contigs differently expressed between male/female

h) Cellular compartment enriched for contigs differently expressed between male/female

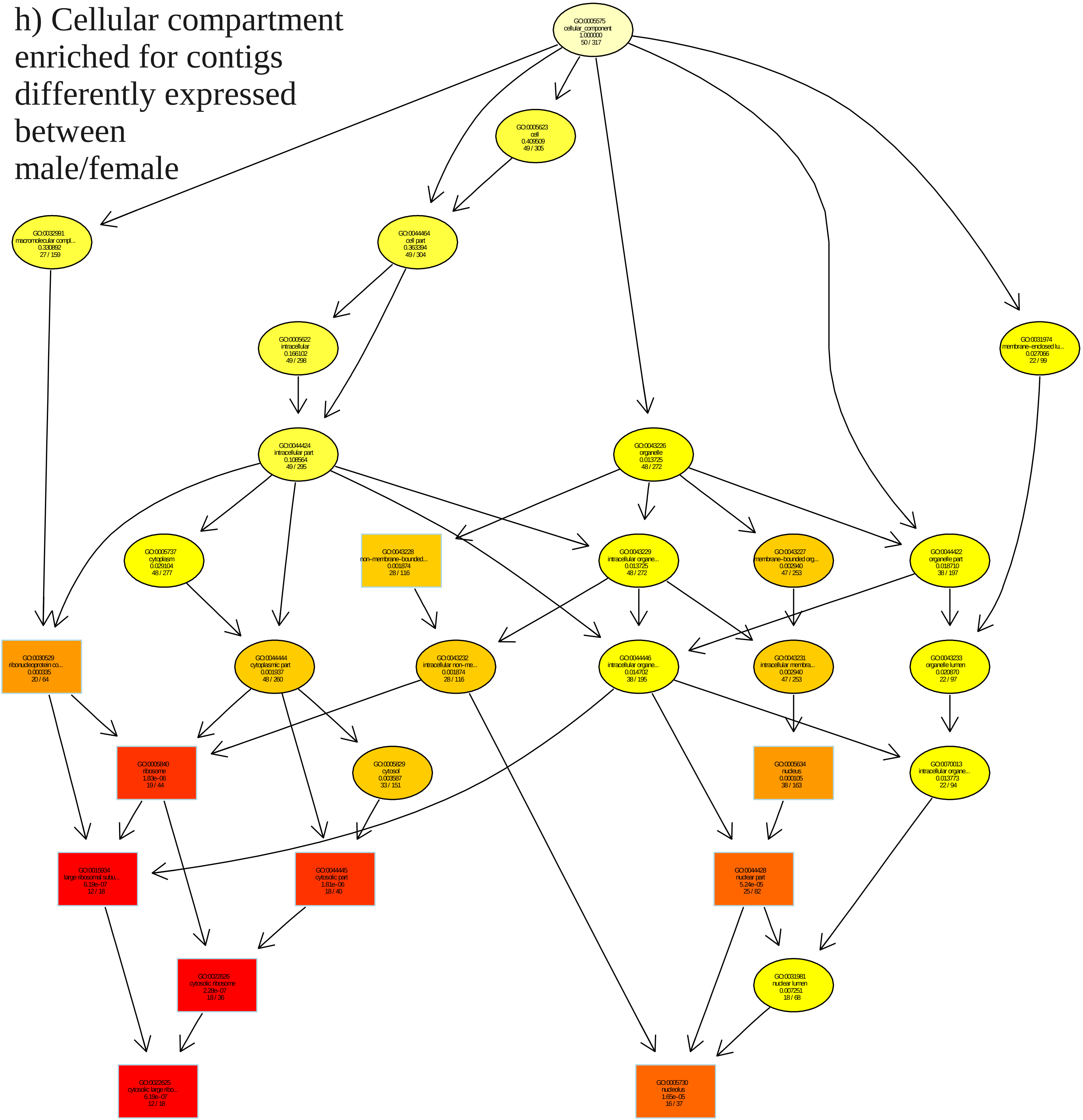

i) Molecular function enriched for contigs differently expressed between male/female

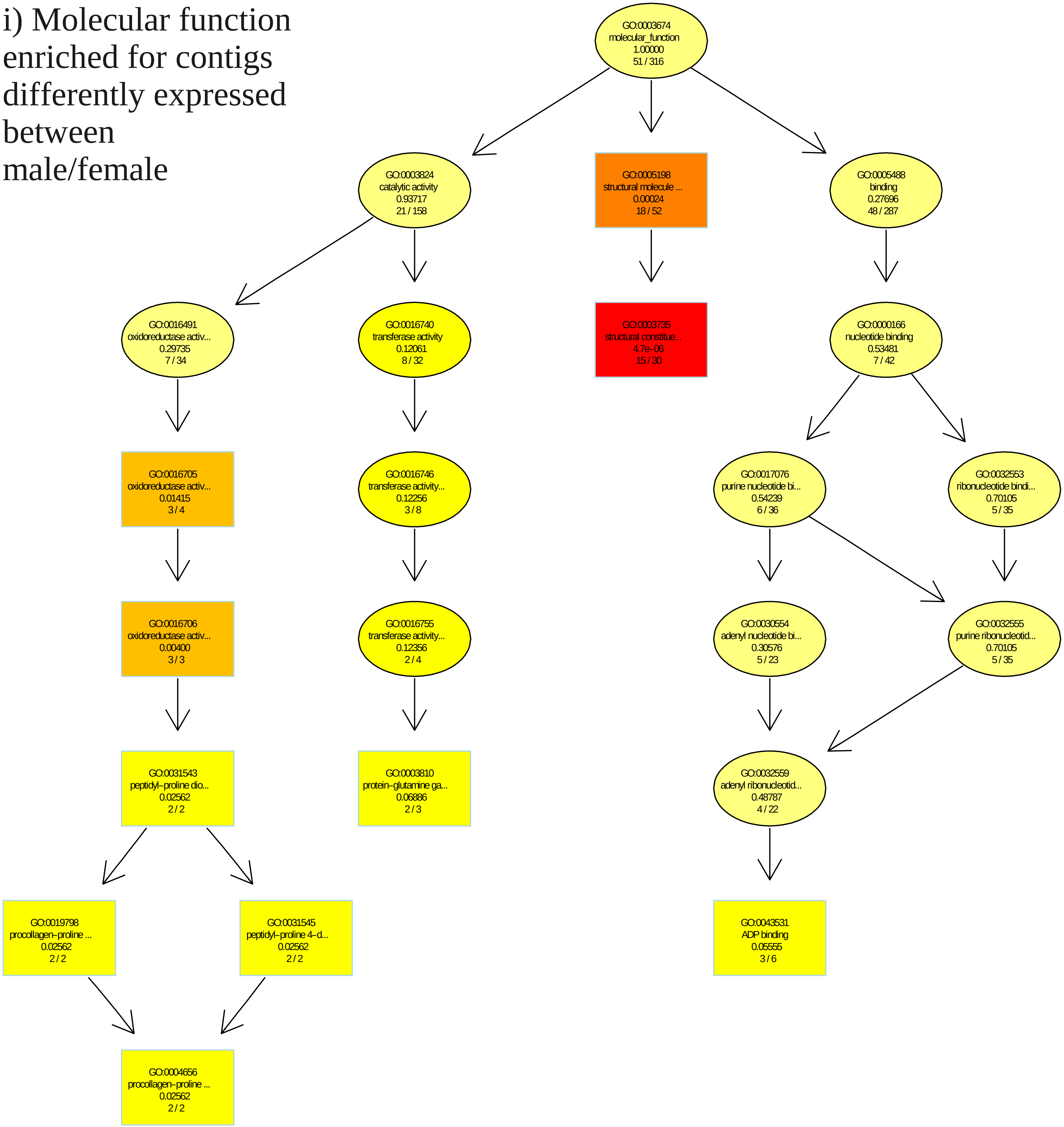

Supplement: Additional file 5 — Additional figure 11 (a-i). Subgraphs of GO induced by the top 10 terms identified as enriched in different sets of genes. Subgraphs of the GO ontology categories induced by the top 10 terms identified as enriched in different sets of genes. Boxes indicate the 10 most significant terms. Box colour represents the relative significance, ranging from dark red (most significant) to light yellow (least significant). In each node the category-identifier, a (eventually truncated) description of the term, the significance for enrichment and the number of DE / total number of annotated gene is given. Black arrows indicate an “is-a” relationship. GO ontology category and the set of genes analysed for the enrichment are indicated in each figure. [file 1471-2164-14-87-S5.pdf]
